# Dopamine and GPCR-mediated modulation of DN1 clock neurons gates the circadian timing of sleep

**DOI:** 10.1073/pnas.2206066119

**Published:** 2022-08-15

**Authors:** Matthias Schlichting, Shlesha Richhariya, Nicholas Herndon, Dingbang Ma, Jason Xin, William Lenh, Katharine Abruzzi, Michael Rosbash

**Affiliations:** ^a^Department of Biology, Howard Hughes Medical Institute, Brandeis University, Waltham, MA 02451;; ^b^Department of Biology, College of Science, Northeastern University, Boston, MA 02136

**Keywords:** dopamine, modulation, circadian, sleep, clock neurons

## Abstract

The metronome-like circadian regulation of sleep timing must still adapt to an uncertain environment. Recent studies in *Drosophila* indicate that neuromodulation not only plays a key role in clock neuron synchronization but also affects interactions between the clock network and brain sleep centers. We show here that the targets of neuromodulators, G Protein Coupled Receptors (GPCRs), are highly enriched in the fly brain circadian clock network. Single-cell sequencing indicates that they are not only enriched but also differentially expressed and contribute to clock neuron identity. We generated a comprehensive guide library to mutagenize individual GPCRs in specific neurons and verified the strategy by introducing a targeted sequencing approach. Combined with a behavioral screen, the mutagenesis strategy revealed a role of dopamine in sleep regulation by identifying two dopamine receptors and a clock neuron subpopulation that gate the timing of sleep.

## Significance

Neuromodulation is essential for adaptive animal behaviors among other physiological processes. It is essential to reliably manipulate neuromodulator pathways to understand their functions in animal physiology. In this study, we generated a CRISPR-Cas9-based guide library to target every G-Protein Coupled Receptor (GPCR) in the *Drosophila* genome and applied it to the well-studied clock neuron network. Notably, these GPCRs are highly enriched and differentially expressed in this small network, making it an ideal candidate to investigate their function. We cell-type specifically mutated GPCRs highly efficiently with no background gene editing detected. Applying this strategy to a specific node of the clock network revealed a role for dopamine in prolonging daytime sleep, suggesting network-specific functions of dopamine receptors in sleep-wake regulation.

Animal function requires communication between different brain centers. An important part of this communication is neurotransmission, which takes place at synapses and leads to the excitation and/or inhibition of downstream neurons. Recent studies stress the importance of a second means of communication, namely, neuromodulation, in the central nervous system. In contrast to classical neurotransmission, neuromodulators do not directly lead to the opening of ion channels but alter second messengers, which then affect electrophysiological responsiveness (1, 2).

Most neuromodulators interact with G Protein Coupled Receptors (GPCRs) (3). This class of receptors is located within the plasma membrane, has seven transmembrane domains, and reacts to a variety of stimuli, including neuropeptides, biogenic amines, and even light. In an inactive state, many receptors are coupled to a heterotrimeric G protein consisting of alpha, beta, and gamma subunits. Depending on their identity, these proteins dissociate upon receptor activation and lead to increases in Ca^2+^ or cAMP or to the activation of transcription via the rho pathway (3, 4). Given these broad ways in which neuromodulators can influence a cell, it is not surprising that they can influence different events. For example, reduced dopamine levels lead to hypoactivity, extended sleep time, or learning deficits, which are phenotypes that often become more extreme with age (5). Notably, the action of dopamine depends on the expression of different GPCRs. Dop1R1 and Dop1R2 are coupled to G_S_ and lead to increases in cAMP, whereas Dop2R is coupled to an inhibitory G protein (G_i_) (6). Similarly, most GPCRs directly influence the molecular properties of individual neurons and are therefore important for the function of many brain circuits.

The circadian clock neuron network (CCNN) of *Drosophila melanogaster* is an ideal model to study the contribution of GPCR signaling to a single adult brain circuit. This network forms a unit controlling locomotor activity in both light-dark (LD) and constant dark (DD) conditions. In LD, the clock generates a bimodal locomotor activity with activity peaks in the morning and the evening and little activity during midday (siesta) and at night. Although this behavior continues in DD, it is less pronounced and has a circadian period that deviates slightly from the normal 24 h.

The fly brain CCNN only consists of 150 neurons, and diverse modes of interactions and distinct functions have been assigned to specific cells. Moreover, several studies emphasize the abundance of circadian neuropeptides and their importance to the functions of this network (7–12). Pigment dispersing factor (PDF) is a key clock neuropeptide and is expressed in two sets of lateral neurons, namely, the four large-ventrolateral neurons (lLNvs) and the four small ventrolateral-neurons (sLNvs) (13); they are important for arousal and morning activity, respectively (14–17). PDF is believed to synchronize its downstream targets, and a loss of the peptide leads to arrhythmicity in DD (7, 18). Three of the six dorso-lateral neurons (LNds) and the fifth sLNv serve a different function and are important for evening activity (14, 15). A pair of dorsal neurons (DN2s) are essential for temperature preference rhythms (19). No function has been assigned to the DN3s, perhaps due to the lack of a specific driver. The DN3s are the most numerous clock neuron group (35 to 40 neurons/hemisphere).

The second most numerous clock neuron group is the DN1 cluster, which consists of ∼15 neurons per hemisphere (19). Under DD conditions, accelerating or decelerating the clock in these cells has no effect on rhythmic behavior, and DN1p output is even dispensable for rhythmic DD behavior (20–23). However, changing the speed of these cells under LD cycle conditions shifts the timing of the evening activity peak, indicating DN1p neurons have a conditional role in circadian timing. Moreover, several groups have shown that specific DN1p neurons affect morning as well as evening activity and influence fly sleep, both the amount of sleep and when sleep occurs (24–26). Consistent with a role in sleep promotion and/or maintenance, imaging and tracing experiments identified physiological pathways connecting specific DN1ps to fly brain sleep regions like the ellipsoid body (24).

Despite a general agreement on the variety of functions carried out by DN1ps, there are discrepancies in assigning specific behavioral roles to specific DN1p subgroups. For example, glutamatergic DN1ps have been identified as controlling the morning component of behavior, whereas another study suggests that these same neurons control evening activity (25, 26). A likely explanation is that the DN1ps are even more diverse than previously thought, i.e., there may be multiple glutamatergic DN1p subgroups. Indeed, a recent single-cell sequencing study found that there are at least 5 different glutamatergic DN1p subgroups, which is consistent with the notion that different DN1p functions might derive from different neuron subpopulations (27). The physiological and anatomical heterogeneity of the CCNN neuron population is probably due to its striking transcription factor specificity as well as to other differences in gene expression between individual neurons (27).

To address the contribution of DN1ps and neuromodulation to sleep behavior as a specific physiological process, we combined the power of single-cell RNA sequencing(scRNAseq) data with cell-specific CRISPR-Cas9-mediated gene mutagenesis. The scRNAseq results not only show that GPCRs are strongly enriched in the CCNN but also that they are highly differentially expressed. Indeed, clustering the clock neurons only based on GPCR expression shows that each subcluster expresses a unique combination of receptors, suggesting that it enables specific network nodes to integrate and respond to different stimuli. To mutate specific receptors in a cell-specific manner, we generated a CRISPR-Cas9-based guide library for all GPCRs following the pioneering work of Port and Bullock (28). We and others had previously demonstrated that this strategy efficiently removes PER or TIM expression from the clock network in a cell-specific manner (29, 30). Here, we also added an adapted targeted genomic sequencing approach to show that the library effectively mutates GPCRs in a cell-specific manner, indicating that the library and strategy constitute a key asset for investigating neuromodulation. Indeed, we used the library in a behavioral screen that identified several GPCRs that promote sleep from within the CCNN. In addition to already known sleep-promoting GPCRs, we discovered that two dopamine receptors (Dop1R1 and Dop1R2) in the DN1ps prolong daytime sleep. Moreover, combining transsynaptic tracing techniques with scRNAseq data shows that different subsets of DN1ps contribute differentially to sleep. The results taken together indicate that dopamine regulates sleep timing via a novel cellular subcircuit within the CCNN that is highly context specific. These findings and methods will facilitate investigating the complex contribution of dopamine as well as other neuromodulators and neurotransmitters to many other physiological processes.

## Results

### Circadian Neurons Show Enriched Expression of Signaling Molecules.

Recent work implicates clock neuron interactions as essential for molecular and behavioral rhythms. For example, manipulating clock neuron subpopulations can affect behavioral timing, and residual clock protein expression in only a few circadian neurons is sufficient to retain some circadian functions (11, 23, 29–31). To identify molecules within the clock network that contribute to the implied network synchrony, we isolated and sequenced fluorescence-activated cell sorting (FACS)-sorted clock neurons (*clk856 > EGFP*) under LD conditions at two times, namely, Zeitgeber time 2 (ZT2) and ZT14, and compared the transcriptomes to those from panneuronal adult head samples (*nSyb > EGFP*).

**Fig. 1.**
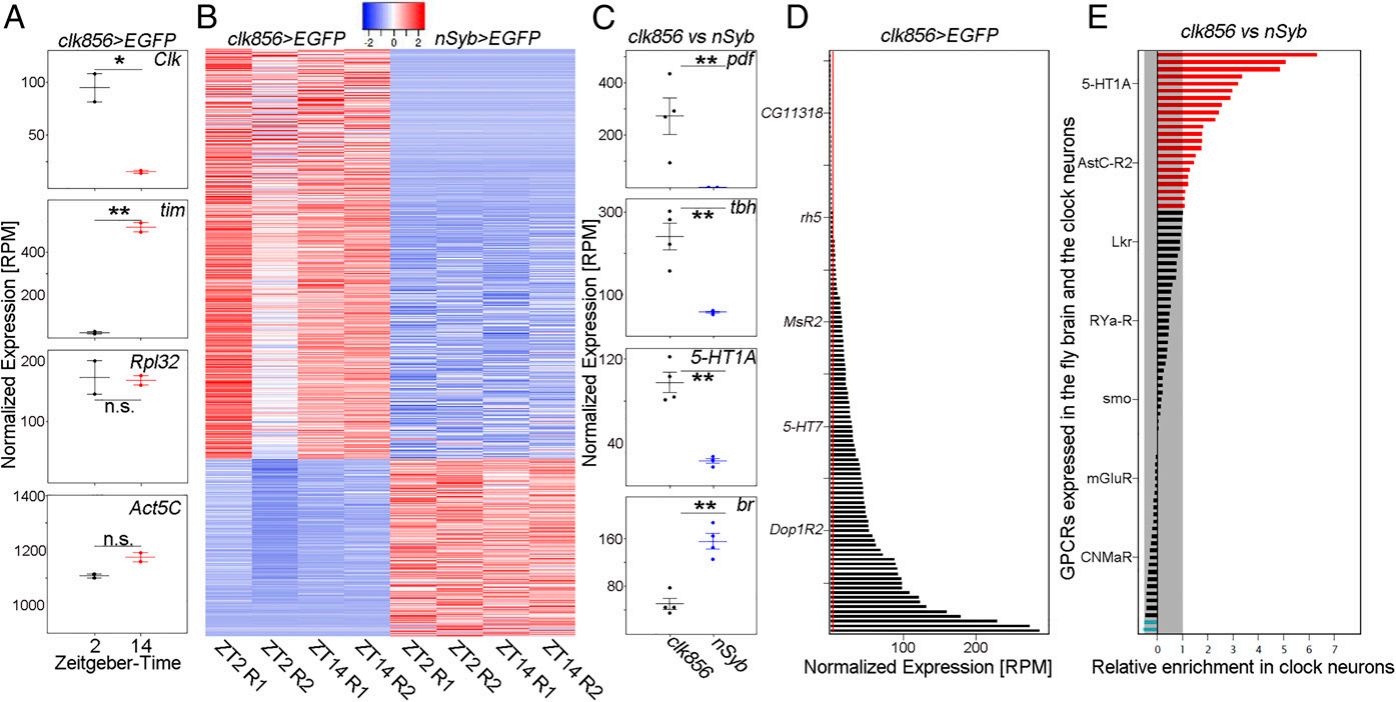
Clock neurons show an enriched expression of GPCRs. (*A*) Plots show normalized expression levels of indicated genes from sorted clock neurons (*clk856 > EGFP*) at ZT2 and ZT14. As expected, *Clk* expression is significantly higher at ZT2 than at ZT14, whereas *tim* expression is significantly higher at ZT14 compared to ZT2. Housekeeping genes such as *Rpl32* and *Act5C* were not significantly different between those timepoints. (*B*) Heatmap of differentially expressed genes between clock neurons (*clk856 > EGFP*) and randomly chosen neurons (*nSyb > EGFP*) at ZT2 (2 repeats, R1 and R2) and ZT14 (2 repeats, R1 and R2) with an at least twofold difference in expression at a false discovery rate of <0.05. Relatively high expression is displayed in red and relatively low expression is displayed in blue (Z-scores indicated on top of the graph). (*C*) Examples of up- or down-regulated genes representing enriched GO terms. *Pdf* is significantly up-regulated in the clock network (GO: Circadian control of sleep wake cycle) as are *tbh* (GO: Octopamine and tyramine signaling pathway) and *5-HT1A* (GO: G protein coupled receptors). *br* on the other hand is significantly down-regulated in the clock network (GO: Antennal development). Both timepoints were combined for this analysis. (*D*) Normalized expression (RPM) of GPCR genes in the clock network. GPCR gene expression varies dramatically between receptors from being not expressed (<3 RPM, red line) to being highly expressed. Both timepoints were combined for this analysis. (*E*) Relative expression of GPCRs in clock neurons (*clk856 > EGFP*) relative to randomly chosen neurons (*nSyb > EGFP*). Reads for individual GPCRs were pooled across timepoints and replicates and a ratio was calculated. A total of 22 GPRs are at least twofold higher expressed in the clock cells (red bars) compared to only 2 GPCRs being at least twofold down-regulated in the clock cells (blue bars). The x-axis is log2 scaled. Both timepoints were combined for this analysis.

We first compared clock gene expression between the time points in the clock neurons. Consistent with expectation (32), *clk* mRNA levels were ∼6× higher at ZT2 than at ZT14, whereas *tim* mRNA levels were 22× higher at ZT14 compared to ZT2 (Fig. 1*A*). Housekeeping genes such as *Act5C* and *Rpl32* were not different between time points (Fig. 1*A*). Not surprisingly, clock gene expression was dramatically lower in the *nSyb* dataset (averaged reads of all *nSyb* libraries show 1,428× reduced *clk* and a 27× reduced *tim* expression compared to sorted *clk856*-neurons; *SI Appendix*, Fig. S1), demonstrating highly efficient enrichment of clock neurons by *clk856 > EGFP* purification.

To address clock neuron gene enrichment more generally, we used edgeR (33) to perform differential gene expression analysis. It identified a total of 1,719 genes as differentially expressed with a false discovery rate of <0.05 between *nSyb* and clock neurons. Of these, 704 genes were significantly up-regulated, and 305 genes were significantly down-regulated in the clock network with at least a twofold change in amplitude (Fig. 1*B*). A Gene Ontology (GO)-term analysis of clock-enriched genes resulted in enrichment in expected categories like the circadian regulation of temperature homeostasis (27-fold), positive regulation of circadian sleep/wake cycle (16-fold), and circadian regulation of gene expression (11-fold). Interestingly, genes associated with signaling pathways were also found to be comparably enriched among the clock-enriched genes and include the octopamine and tyramine signaling pathways (16-fold), the serotonin receptor signaling pathway (16-fold), and the GPCR signaling pathway (12-fold). Other unrelated pathways are enriched in the *nSyb* neurons relative to the clock neurons (for an example, see Fig. 1*C*). The increased expression of genes involved in intercellular signaling pathways, including serotonin signaling and GPCRs, strongly implicate neuronal communication in clock network function.

### GPCRs Are Differentially Expressed within the Clock Neuron Network.

The enrichment of GPCR signaling pathways inspired a focus on quantitative features of GPCR expression. Surprisingly, more than two-thirds of the 124 GPCR mRNAs encoded by the *Drosophila* genome are expressed in the clock neurons (reads per million [RPM], >3; Fig. 1*D*) with an almost 100× difference between the least and most expressed of these 86 mRNAs. The *nSyb* data had similar differences in GPCR expression levels (*SI Appendix*, Fig. S2). A direct comparison with the *nSyb* dataset indicates that 22 GPCRs are at least twofold up-regulated in the clock network, whereas only 2 receptors are down-regulated (Fig. 1*E*).

**Fig. 2.**
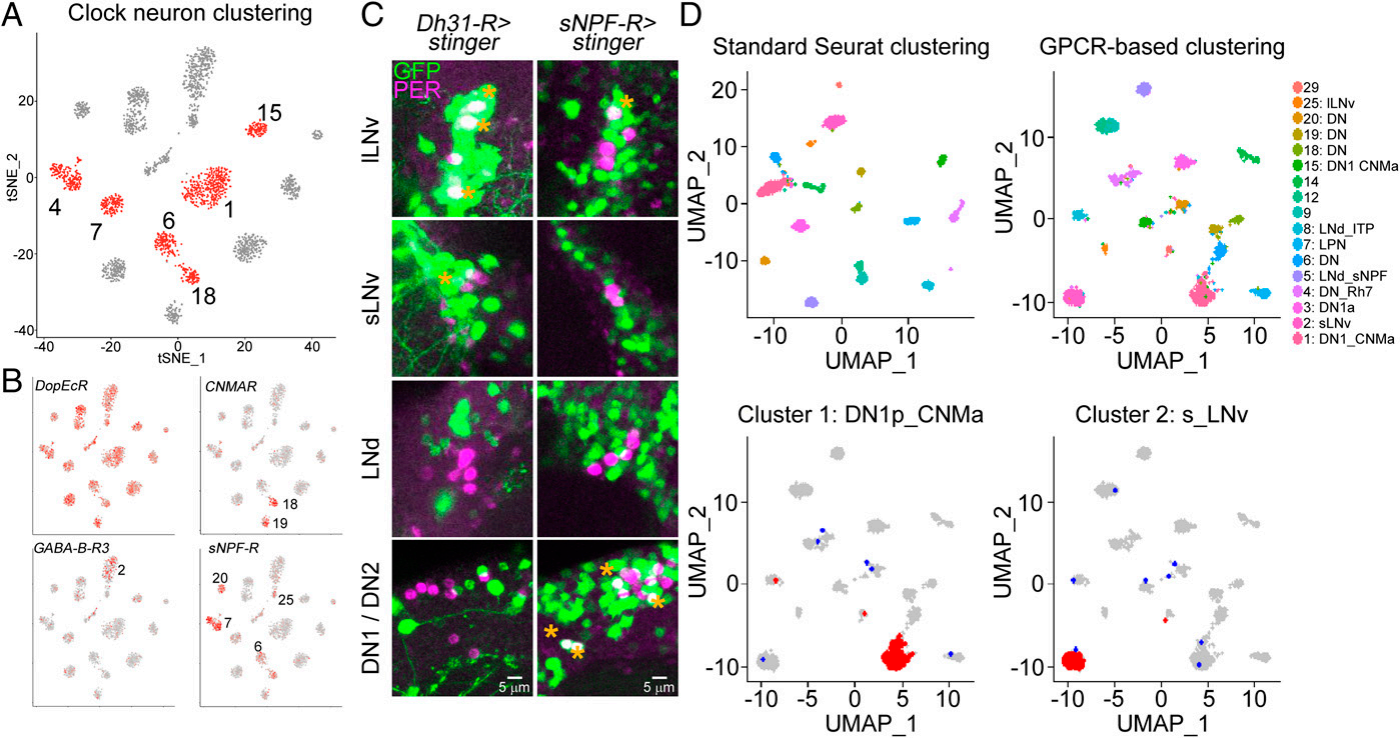
GPCRs are differentially expressed and can define clock neuron identity. (*A*) scRNAseq of clock neurons (*clk856 > EGFP*) identifies 17 bona-fide clock neuron clusters. DN1 neurons can be separated into six different clusters (1, 4, 6, 7, 15, and 18, labeled in red). For details on clustering see ref. 27. (*B*) Expression of different GPCRs within these 17 clusters. *DopEcR* is highly expressed in all clock neuron clusters, whereas *CNMaR*, *GABA-B-R3,* and *sNPF-R* are more differentially expressed. For details see "GPCRs are differentially expressed within the clock neuron network" section. (*C*) Immunohistochemistry of whole-mount brains stained against GFP (green) and PER (magenta). Nuclear GFP (UAS-*stinger*) was expressed under the control of GAL4-knock-in lines. *Dh31-R-GAL4* drives expression in three out of four lLNvs and one sLNv, and does not express in DN1s, DN2, or LNds. *sNPFR-GAL4* is expressed in 1 lLNv, 2 DN1s, and the DN2 neurons. (*D*) Seurat clustering of individual neurons included in 17 high-confidence clusters. Unsupervised clustering (*Top Left*) recapitulates previously published results. Similarly, clustering only based on GPCR expression generated 17 different clusters (*Top Right*). *Bottom* row: Analysis of retained (red) or newly assigned (blue) clock neuron identities when plotted only based on GPCRs. Most neurons in clusters 1 (*Bottom Left*) and cluster 2 (*Bottom Right*) are assigned the same clusters (red dots), whereas only a few cells were assigned to different clusters (blue dots) based on GPCR-only clustering of clock cells.

The RNA expression data suggest that many individual GPCRs are either expressed at significantly higher levels in all clock neurons or are predominantly expressed in a few neuronal subpopulations. To address these alternatives, we analyzed previous scRNAseq data for GPCR expression and focused on the 17 high-confidence clock neuron clusters (Fig. 2*A*); these clusters are missing most of the enigmatic DN3 clock neurons but include most if not all well-characterized clock neurons including all lateral and most dorsal clock neurons.

The dopamine receptor *DopEcR* transcript is highly expressed in the *nSyb* as well as the *clk856* dataset with high transcript levels in all clock neuron clusters (Fig. 2*B*). The differential expression patterns of more poorly expressed GPCR transcripts within the clock neuron population were similarly impressive. For example, *CNMaR* appears to be almost exclusively expressed in one DN1p and the DN2 cluster, whereas *Gaba-B-R3* is highly enriched in the sLNv cluster. sNPFR is expressed more broadly but still mostly in the clusters defining the DN1ps, the lLNvs, and the DN3s (Fig. 2*B*). A differential GPCR expression pattern is also apparent when comparing all GPCR expression using scRNAseq despite dramatic variation in expression levels (*SI Appendix*, Fig. S3).

To confirm this cell specificity in an independent way, we used GAL4 lines in which the GAL4 sequence was integrated into endogenous GPCR-expressing loci and used to express nuclear GFP (*UAS-stinger*) (34). The overall expression levels of individual GPCRs correlated nicely with the *nSyb* sequencing data (*SI Appendix*, Fig. S2). For example, some GPCRs such as the aforementioned *DopEcR* appear to be expressed almost panneuronally within the brain, whereas others are not expressed in the brain or only in one to two cells per hemisphere (*SI Appendix*, Fig. S2).

To detect the expression of specific GPCRs within the clock neuron network, we focused on GPCRs with striking expression patterns from our single-cell data and determined the overlap of PER and GFP by costaining knock-in lines with anti-PER. The single-cell data indicate that *DH31-R* is highly enriched in the lLNvs and slightly enriched in the sLNvs and the DN1ps. The GAL4-knock-in line is strikingly consistent with this pattern; three out of four lLNvs were GFP-positive, whereas only one out of four sLNv neurons was labeled, explaining the differences in expression levels. As predicted, no LNds were labeled, but we could also not detect GFP expression in the DN1 neurons in most brains. Similar cell type specificity was also observed with *sNPFR*, which mainly labeled DN1 neurons and one out of four lLNvs (Fig. 2*C*). The data taken together suggest that GPCRs are strongly differentially expressed within the clock network. Notably, differences appear even between individual cells within an anatomically mostly uniform cluster like the lLNvs (*SI Appendix*, Fig. S4).

Might these GPCR expression differences be sufficient to define clock cell identity within the clock network? We originally identified 17 high-confidence clock neuron clusters based on common highly variable genes across different time points and conditions (27). Here, we first used Seurat, an unsupervised clustering method, and reproduced the previously published 17 high-confidence clock neuron clusters (Fig. 2*D*). We then used GPCR expression alone for clustering, omitting the contribution of all other genes expressed in the individual cells. This unsupervised GPCR-based clustering also identified 17 distinct clusters (Fig. 2*D*). To our surprise, many of these newly generated clusters could be mapped onto the previously published dataset (27), indicating that the GPCR-generated clusters are of biological and anatomical significance. For example, the newly identified cluster 1 includes ∼90% of this previously identified sLNv cluster. This is also the case for other clock clusters like the DN1s (Fig. 2*D*). The data therefore indicate that GPCR expression alone is sufficient to define the identify of most clock neurons and also suggest that each cluster may exhibit a unique neuropeptide response pattern.

### A Guide Library Allows for Cell-Specific Manipulations of All GPCRs.

The likely contribution of GPCRs to clock neuron identity and the neuropeptide requirement for clock neuron synchrony inspired the development of a general strategy to eliminate any GPCR in a neuron-specific manner. Relevant to this goal, we and others recently showed that CRISPR-Cas9-based mutagenesis is superior to RNA interference (RNAi)-mediated gene expression knockdown in the fly brain (29, 30). As the former also does not require strongly expressing driver lines, it is much more amenable to highly neuron-specific split-GAL4 lines.

**Fig. 3.**
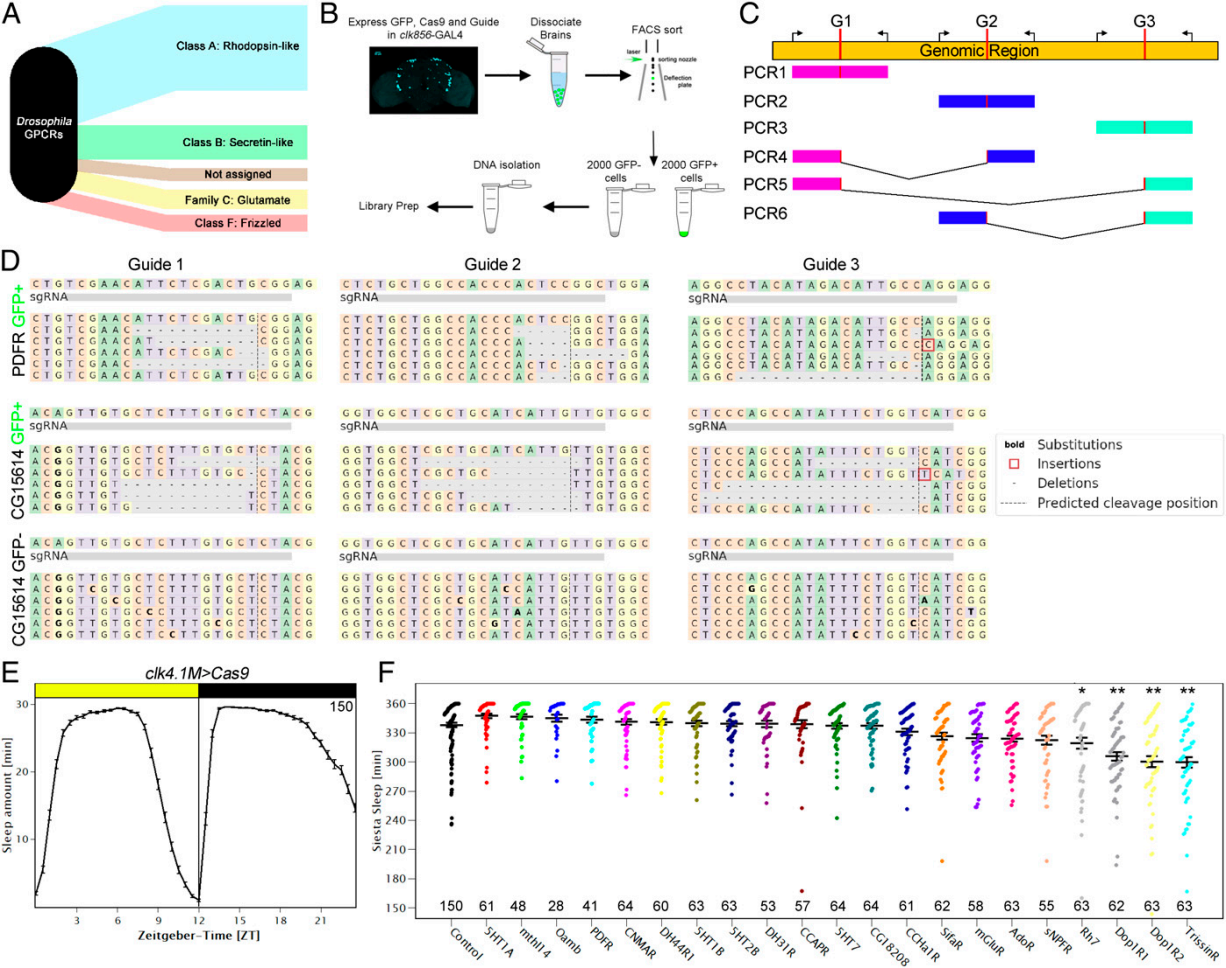
A guide RNA library for all GPCRs of the fly genome allows for cell-specific mutagenesis. (*A*) *Drosophila* GPCRs can be categorized into 5 distinct groups (reviewed in ref. 4) (*B*) Workflow of the targeted genomic sequencing assay. Clock neurons were labeled with EGFP (*clk856 > EGFP*) while expressing Cas9 and the guide RNA of interest which allows for cell-specific manipulations. GFP-positive cells were analyzed for possible mutagenesis. GFP-negative cells were used as controls for cell specificity. (*C*) Schematic of genomic sequencing approach. Each genomic region was targeted by three independent guide RNAs (G1 to G3) that led to double-strand breaks (DSBs) at the desired locations (indicated in red). We simultaneously used three sets of primers for each reaction amplifying ∼250 bp. Depending on the timing of guide-RNA-mediated DSBs, we expect either short deletions (PCR1 to 3) or big deletions (PCR4 to 6) in the event of two guides cutting at the same time. (*D*) Genomic sequencing results of targeted genomic sequencing. *Top* panel: Sequences of GFP-positive cells of *clk856 > EGFP, Cas9, PDFR-g* flies. All three guides are able to cause small deletions at the expected site of DSB. *Middle* panel: Sequences of GFP-positive cells of *clk856 > EGFP, Cas9, CG15614-g* flies. All three guides are able to cause small deletions at the expected site of DSB. *Lower* panel: Sequences of GFP-negative cells of *clk856 > EGFP, Cas9, CG15614-g* flies. No deletions were detected in GFP-negative cells. (*E*) Sleep behavior of control flies ± SEM expressing *Cas9* in DN1 clock neurons (*clk4.1M > Cas9*). The flies showed the expected sleep pattern with low sleep in the morning and evening and high levels of sleep at night and during the siesta indicating that expressing Cas9 in the dorsal neurons does not affect normal sleep behavior. *n* = 150. (*F*) Sleep amount of flies with mutated GPCRs during the siesta (ZT3 to 9) in LD. *Clk4.1M > Cas9* flies were used as controls, for experimental flies, *Clk4.1M > Cas9* flies were crossed to UAS-guide-RNA lines as indicated on the x-axis. Four guide RNAs significantly reduced sleep during the siesta. Numbers indicate the number of flies used for the behavioral screen. Statistical analysis was performed using a one-way ANOVA followed by a post hoc Tukey pairwise comparison. *P < 0.05 and **P < 0.01 compared to control flies.

The *Drosophila* genome encodes 124 GPCRs. They are like mammalian GPCRs and react to a variety of stimuli, including biogenic amines, neurotransmitters, neuropeptides, and even light (Fig. 3*A*) (4). To mutate these receptors, we generated UAS-guide lines, each of which expresses three guides targeting the coding sequence of an individual GPCR. Three guides have previously been shown to efficiently mutate eye tissue and provide high mutagenesis efficiency by compensating for potential noncutting guides (28); this strategy also worked well to remove PER and TIM from the clock system (29, 30). We used *clk856*-GAL4 to drive the expression of GFP, Cas9, and the guides of interest to mutate individual GPCRs in most of the clock network. The goal was to generate double-strand breaks within the coding sequences of GPCR genes. Given that repair is error prone, this strategy should generate small deletions of variable sizes and frame shifts, resulting in nonfunctional GPCRs.

As there are no reliable antibodies for many of the receptors, we verified the strategy with a targeted genomic sequencing approach. As the clock neurons were simultaneously labeled with GFP as well as mutated by the CRISPR-Cas9 system, we FACS sorted and analyzed 2,000 GFP-positive cells, which should have been mutated, and 2,000 GFP-negative cells, which should remain wild type (Fig. 3*B*). We designed three sets of primers flanking each of the guide binding sites to allow for gene-specific amplification. As three guides are being used at the same time, there can either be small deletions in the area of guide binding (Fig. 3*C*, PCR1 to 3) or larger deletions if multiple guides cut at the same time, resulting in different DNA fragment combinations (Fig. 3*C*, PCR4 to 6). We analyzed five randomly chosen target genes on three different chromosomes (*PDFR* and *Tre1* on the X-chromosome, *mAchR-A* and *CG15614* on the second chromosome, and *CrzR* on the third chromosome) to avoid possible biases from chromosome location. *PDFR* served as a positive control; guide-mediated mutagenesis of the clock network with its guides completely reproduced *PDFR* full body mutant (*han^5304^*) phenotypes (35, 36).

All three *PDFR* guides generated deletions of variable sizes at the predicted cut sites in GFP-positive cell DNA (Fig. 3*D*). Moreover, there were large deletions as indicated by a genomic fragment representing a deletion of several thousand base pairs (Fig. 3*C*, PCR5). GFP-negative cells in contrast showed no deletions in the investigated area, suggesting that there is no background mutagenesis due to leaky expression in nontarget cells. We then assayed *CG15614*. Like for *PDFR*, all three guides generated deletions, whereas GFP-negative cells were unaffected (Fig. 3*C*). *Tre1* and *CrzR* had similar results, whereas only two out of the three guides for mAchR-A created deletions (*SI Appendix*, Fig. S5).

The efficacy of the three individual guide sequences to generate small deletions varied substantially, from between 0.7% (as mentioned above for one guide of *mAchR-A*) to more than 50%, with no evident chromosome or location bias. When an individual guide failed to generate mutations, the other two guides efficiently mutated the gene of interest; this indicates the importance of using several guides to compensate for potential noncutters. There was also a reduced frequency of bigger deletions of coding sequences. For example, two genes (*CrzR* and *mAchR-A*) did not show big deletions, whereas 2% to 6% of the reads from other genes reflected big deletions. It is important to note that these percentage are based on the sequencing of pooled neurons from several animals and therefore do not reflect a single, mutated neuron. Nonetheless, the collective data support the original assertion based on eye color essays (28), namely, that combining three guides per gene is an effective strategy to manipulate genes of interest.

### DN1p Modulation Alters the Sleep Structure of Male Flies.

To exploit this functional library, we focused on the DN1ps. This specific group of dorsal neurons influences several aspects of *Drosophila* behavior. This is because manipulating these cells affects activity in the morning and during the siesta as well as in the evening. In addition, DN1ps affect sleep and connect to sleep centers within the central complex (24–26). Recent work also showed that this group of neurons can be subdivided into six independent clusters with functions that are not yet clearly understood. The molecules that affect sleep within these neurons are also mostly unknown. To address this question, we used *clk4.1M*-GAL4, which expresses in 8 to 12 of the 15 DN1ps per hemisphere. We first reproduced previous experiments; activating these neurons significantly altered sleep in the middle of the day, the prominent siesta of male flies (*SI Appendix*, Fig. S6).

To identify candidate DN1p GPCRs, we turned to our single-cell data and identified 21 GPCRs enriched in DN1ps compared to the other clock cells. We then performed a behavioral screen in which we compared the behavior of control flies with flies harboring mutated GPCRs in their DN1ps. The control flies expressed *Cas9* but no guides in these neurons (*clk4.1M > Cas9*).

As expected, *Cas9* expression in the DN1ps did not affect fly behavior; they showed the canonical sleep pattern with consolidated sleep at night and during the siesta with almost no sleep in the morning and the evening; this reflects the standard bimodal activity pattern (Fig. 3*E*). We then focused on the siesta and quantified sleep levels between ZT3 and ZT9. Male flies sleep extensively during this time, leading to a median of ∼5.5 h of siesta sleep in control flies. Of the 21 mutated strains, 4 significantly reduced their siesta sleep compared to the control group by one-way ANOVA followed by a post hoc Tukey test (Fig. 3*F*). One GPCR is *rh7*, which reproduces known whole-body mutant phenotypes (37). Another is *TrissinR*, which reduced sleep by half an hour. Its relevant ligand, Trissin, is expressed in two LNds, suggesting that intraclock neuron communication, LNd to DN1p, is relevant to sleep regulation (27). Eliminating two different dopamine receptors, namely, Dop1R1 and Dop1R2, also reduced siesta sleep, each by approximately half an hour.

### Dopaminergic Input to DN1 Neurons Reduces Siesta Sleep.

**Fig. 4.**
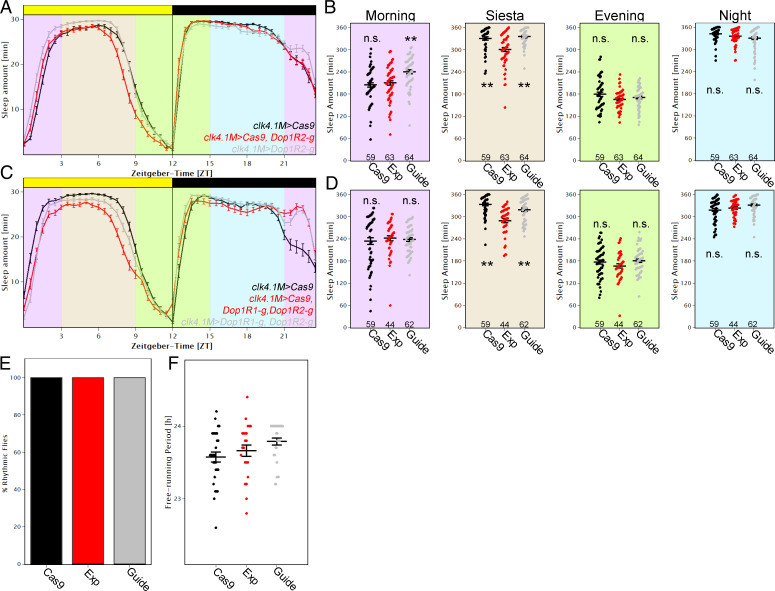
Modulation of DN1ps by dopamine enhances sleep. (*A*) Average sleep profile of male flies in which *Dop1R2* was mutated in the DN1ps (red) and controls (gray and black). Error bars represent SEM. Background colors indicate 4× 6-h periods that were quantified in *B*. (*B*) Quantification of sleep separated into four different time zones, as follows: morning (ZT21 to ZT3), siesta (ZT3 to ZT9), evening (ZT9 to ZT15), and night (ZT15 to ZT21). Removing *Dop1R2* in the DN1ps significantly reduced sleep during the siesta (one-way ANOVA followed by post hoc Tukey test shows significant differences between EXP and both controls, *P* < 0.01 each), whereas other times of day were not affected. (*C*) Average sleep profile of male flies in which *Dop1R2* and *Dop1R1* were mutated in the DN1ps (red) and controls (gray and black). Error bars represent SEM. Background colors indicate 4× 6-h periods that were quantified in *D*. (*D*) Quantification of sleep separated into four different time zones. Removing *Dop1R2* and *Dop1R1* in the DN1ps significantly reduced sleep during the siesta (*P* < 0.01), whereas other times of day were not affected. n.s., not significant. (*E* and *F*) Flies with mutated *Dop1R1* and *Dop1R2* in the DN1ps (Exp) have rhythmicity the same as controls (*E*) and no effect in free-running period (*F*) when recorded in DD compared to both controls (*clk4.1M > Cas9* [black] and c*lk4.1M > DopiR1-g, Dop1R2-g* [gray]).

The sleep reduction by mutating Dop1R1 and Dop1R2 in the DN1ps was surprising, as dopamine is traditionally activity promoting (38–40). We therefore addressed this sleep effect in more detail by dividing the 24-h day into four equal sections of 6 h each, as follows: ZT21 to 3 for morning sleep, ZT3 to 9 for siesta sleep, ZT9 to 15 for evening sleep, and ZT15 to 21 for nighttime sleep (Fig. 4*A*). As expected, flies slept more during the siesta and at night compared to morning and evening (Fig. 4*B*). Quantifying sleep with *Dop1R2* mutated in DN1ps showed that sleep was only significantly reduced during the siesta in the experimental flies (*P* < 0.01) compared to the Cas9 (*clk4.1M > Cas9*) and guide (*clk4.1M > Dop1R2-g)* control flies. Mutating *Dop1R1* in the DN1ps had very similar effects (*SI Appendix*, Fig. S7).

Will mutating both *Dop1R1* and *Dop1R2* at the same time have an even stronger effect on sleep? Although siesta sleep is still significantly reduced compared to both control strains with no effect at any other time of day, we did not observe any additive effect; the double-mutated strain had a similar effect compared to the single mutant strains. (Fig. 4*C* and *D*).

To provide further support for this approach, we expressed *Cas9* and guides against *Dop1R2* in the dorsal fan-shaped body and reproduced the increased siesta sleep phenotype observed by Pimentel et al. (41) using the same dFSB driver *23E10* and RNAi knockdown of *Dop1R2* (*SI Appendix*, Fig. S8). The results taken together indicate that the target receptors and cells dictate the effect of dopamine, which can promote sleep as well as wake.

The sleep patterns indicate that the siesta is terminated earlier in flies with mutated dopamine receptors compared to controls, suggesting that dopaminergic input contributes to timing the end of the siesta. Notable in this context is the traditional *per^S^* mutant strain; it has a very short free-running period with a similar LD phenotype, e.g., the evening peak occurs during the daytime (41, 42). Yet there was no effect of removing both dopamine receptors from the DN1ps on period length or rhythmicity; experimental groups as well as controls were identical to wild-type flies (Fig. 4*E* and *F*), suggesting that changes in clock speed are not responsible for the sleep phenotypes. The data indicate that dopaminergic input onto DN1p neurons enhances sleep during the siesta, likely caused at least in part by delaying the onset of evening activity.

### Subclustering of DN1 Neurons Allows the Differentiation of Neurons Controlling Evening Activity.

How might dopaminergic modulation of DN1ps impact the siesta? To address this question, we first used the *trans*-Tango technique to label target neurons through its anterograde transsynaptic circuit tracing (43). We applied this technique to the dopaminergic system (*TH*-GAL4) and labeled downstream neurons with GFP. Because there were many GFP-positive neurons, we costained with anti-PER and investigated colocalization of GFP and PER immunoreactivity. The dopaminergic system appears to contact a variety of clock cells including PDF cells, 3 out of 6 LNds, 2 of 2 DN2s and on average 4 of 15 DN1ps, confirming that the DN1s are indeed among the direct downstream partners of TH cells (*SI Appendix*, Fig. S9).

**Fig. 5.**
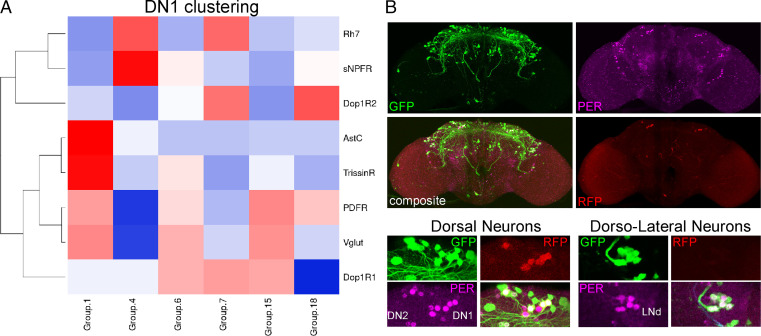
Subclustering of DN1ps indicates their function in controlling evening activity. (*A*) Clustering of DN1ps using key molecules expressed in these cells. (*B*) *trans*-Tango experiment of the *Vglut-DBD*, *per-AD* split-GAL4 driver line stained with anti-GFP (*trans*-Tango positive neurons), anti-PER, and anti-RFP (GAL4- expressing neurons). Several neurons in the dorsal and lateral part of the brain are downstream of the *Vglut*-positive DN1 neurons. Notably, all LNds are included, indicating an anatomical connection of siesta behavior with cells controlling evening activity. For details see discussion.

Examining the 6 DN1p RNA expression clusters in more detail indicated that *Dop1R1* and *Dop1R2* are primarily expressed in 4 clusters, numbers 6, 7, 15, and 18 (Fig. 5*A*). Intriguingly, four is identical to the number of tyrosine hydroxylase neurons targeting DN1ps. Importantly, the size of these four clusters is rather small, indicating that several of them may only contain a single cell (27). We also considered the two remaining DN1p clusters, namely, clusters 1 and 4. The biggest cluster (#1) is the only one that expresses *AstC*. We showed previously that *AstC* is expressed in four DN1 neurons, which fits well with the size of this cluster (10, 27). Knockdown of *AstC* affected the timing of the evening peak in summer or winter days, suggesting that these neurons also contribute to evening activity, at least as a function of different seasons (10). In addition, this is the only cluster expressing *TrissinR,* which also produced a siesta phenotype in our behavioral screen (*SI Appendix*, Fig. S7). The data taken together indicate that several and perhaps most DN1 clusters contribute to evening activity.

We exploited the striking difference in *Vglut* expression between morning and evening DN1p cells to further address how the DN1ps regulate evening activity (Fig. 5*A*). Combining a *per*-AD with *Vglut*-DBD labeled on average seven to eight DN1s per hemisphere, consistent with the expected number of neurons in these clusters (*SI Appendix*, Fig. S10). *Trans*-Tango experiments with this split-GAL4 revealed that these seven to eight DN1p neurons primarily target neurons in the dorsal part of the brain (Fig. 5*B*). They include DN1s, DN2s, and DN3s, and this split-GAL4 also targets all LNds in the lateral part of the brain; they are a major controller of evening activity. These data reinforce previous results (25) indicating that the glutamatergic subset of DN1ps likely controls evening activity and sleep at least in part through their interactions with other clock neurons. Importantly, the upstream influence of dopamine on these cells and their functions widens the influence of the environment and brain state over the siesta and evening cell timing (see *Discussion*).

## Discussion

We show here that GPCRs are strongly expressed in the fly brain CCNN and are capable of identifying individual clock neurons. To identify individual receptors that contribute to specific neuron function, we combined a behavioral-sleep screen with a previously validated CRISPR-Cas9-specific neuron-mutagenesis strategy that exploited comprehensive *Drosophila* GPCR guide library. The strategy was verified with a targeted sequencing approach and revealed a role of dopamine in sleep maintenance during the siesta. Dopamine generally inhibits sleep by stimulating locomotor activity, in flies as well as mammals (38–40, 44). For example, compounds like amphetamine increase synaptic dopamine levels, which enhance fly activity and inhibit sleep (45). However, a specific sleep-promoting subpopulation of DN1ps uses this neurotransmitter for the opposite purpose, namely, to prolong sleep during the siesta. This surprising conclusion resulted from the identification of the two dopamine receptors Dop1R1 and Dop1R2 as well as a clock neuron subpopulation, within which the two receptors gate the timing of daytime sleep.

The CCNN is an ideal platform to study neuromodulation, as genetic studies underscore the importance of neuropeptides to circadian behavior and even to circadian neuron subtype identification (11, 23, 32). This is also true for the mammalian brain and its CCNN, the suprachiasmatic nucleus (46). Notably, most neuropeptides act through GPCRs, and 22 of the GPCRs expressed within the fly brain are at least 2× up-regulated in the CCNN (Fig. 1*E*). This result is even more striking in light of our scRNAseq data, which show that many GPCRs are differentially expressed among clock neurons (Fig. 2).

To verify expression patterns, we costained relevant GAL4 knock-in lines with an anti-PER antibody and thereby determined the overlap between receptor and clock protein gene expression. Despite some minor differences, the knock-in lines were broadly consistent with the RNAseq data and reinforced the notion of cell-specific GPCR expression (Fig. 2*C*). Importantly, individual GPCRs appeared to be expressed in only some cells of supposedly uniform clusters. For example, only one cell of cluster 1 stains for *DH31-R* (Fig. 2*C*), suggesting even more clock neuron diversity than previously indicated (27).

The data underscore more generally the importance of GPCR expression to the CCNN. To investigate this further, we analyzed our single-cell data based only on GPCR expression. There were only marginal changes in cluster formation, i.e., we were still able to generate 17 clusters which were similar to those previously published (27). This surprising result indicates that GPCRs can identify individual neuron subpopulations, at least within the clock system, and suggests that they can define functional identity. This likely includes subtle contributions to phenotype beyond defining cell-specific ligand responses.

GPCRs are difficult to study genetically in mammals. This is because there are usually multiple genes encoding a single GPCR. The situation is simpler in flies where there is usually only one gene that encodes each of the 124 GPCRs encoded in the fly genome. Our guide library targets each of these GPCRs and uses three independent guides for each receptor, a strategy shown to be highly efficient in previous studies (28–30). As there are no available antibodies for GPCRs, we validated the strategy with a targeted genomic sequencing approach from isolated neurons. It can directly characterize the molecular consequences of the guide-mediated mutagenesis on the clock network. Like the guide strategy previously used to eliminate PER (29, 30), the GPCR guides can reliably delete GPCR-encoding genomic DNA from GFP-positive cells with no detectable deletions in GFP-negative cells. This further supports the notion that the UAS constructs impact minimally if at all cells outside of the canonical GAL4 expression pattern (Fig. 3).

We focused on DN1ps because of their molecular complexity as well as their known contributions to morning activity, the siesta, and even nighttime sleep (47). Of the 21 DN1p-enriched GPCRs, 4 promote sleep based on the knockdown results. One of them, *rh7*, reproduced previously published results of whole-body mutations, suggesting that it contributes to the siesta at least in part via DN1p expression (37). To our surprise, two dopamine receptors, namely, *Dop1R1* and *Dop1R2*, also promote siesta sleep via the DN1ps and do so by gating the timing of siesta termination. This role resembles previous results indicating that *Vglut* and *AstC* are expressed in the DN1 neurons and influence the siesta and/or evening activity under conditions that mimic seasonal regulation (10, 25, 48).

There are four different clusters of DN1 neurons that show an elevated expression of *Dop1R1* or *Dop1R2*, suggesting that one or more of these clusters are responsible for the siesta phenotype. Unfortunately, the lack of more narrow drivers precludes more precise identification.

**Fig. 6.**
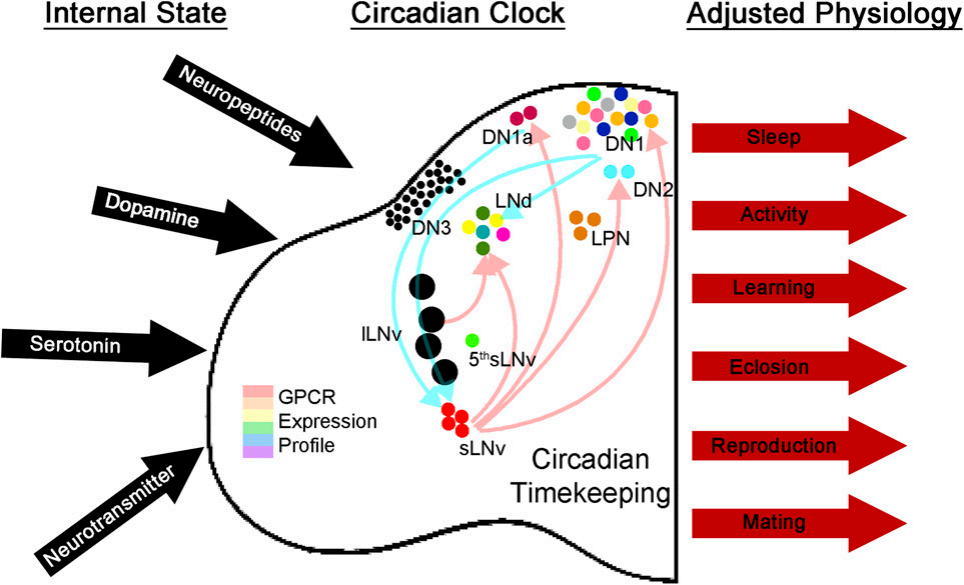
Diversity of GPCR expression among clock neurons as a mechanism for tuning physiology and behavior. The clock neuron network expresses RNAs for ∼two-thirds of all GPCRs encoded by the fly genome. This allows the network to constantly assess the outside environment as well as the state of other networks in the brain that can then signal to the clock center. These signals can then alter the physiology of the clock and lead to changes in clock-controlled output.

How can dopaminergic input influence the circadian gating of siesta sleep? The putative downstream targets of dopaminergic neurons correlate nicely with previously published imaging data; lateral as well as dorsal clock neurons increase their cAMP levels in response to bath-applied dopamine (49). Bath application of the neuropeptide PDF causes a similar cAMP increase (50). Notably, this increase stabilizes PER, which is thought to delay the timing of the molecular clock (51). A similar mechanism might apply to dopamine and the DN1ps, which would then delay clock timing within these cells. Removing dopamine receptors would then lead to a decrease in cAMP levels and a consequent advance in timing, thereby explaining the early termination of the siesta in our experiments. Independent of such mechanistic speculation, our data add to our view of how the clock system works to regulate sleep; dopaminergic input presumably reflects the monitoring by the CCNN of brain and environmental status, which then adjusts circadian timing. This change in CCNN properties likely leads to altered release of neuropeptides and/or neurotransmitters, which will then alter whole animal physiology. (Fig. 6). This complexity could even be part of a resilience and plasticity neuropeptide and neurotransmitter system similar to that described in the crustacean stomatogastric ganglion.

We have found this mutagenesis strategy and guide library to be highly effective and far superior to and more reliable than RNAi. There are no background issues, and weak expression is still sufficient to generate mutations. Given the broad role of neuropeptides, transmitters, and GPCRs in most aspects of brain function and behavior, we anticipate that this GPCR mutagenesis strategy and library will be of use to a broad range of fly brain neuroscientists, well beyond the circadian system and the few other researchers who have already used them (52).

## Materials and Methods

### Fly Strains and Rearing.

Flies used in this study are listed in *SI Appendix*, Table S1. All flies were raised at 25 °C in a temperature-controlled incubator in LD 12:12 h.

### Generation of Fly Lines.

To generate UAS-guideRNA flies, we used the pCFD6 vector (addgene #73915, described in ref. 28). In short, we generated three guides targeting the coding sequence of each GPCR. To identify possible target sites and avoid off-target effects, we used the optimal target finder developed by C. Dustin Rubinstein, Ed O'Connor-Giles, and Kate M. O'Connor-Giles (53). Gene-specific guide sequences were then incorporated into the primers (*SI Appendix*, Table S2), and the protocol described in ref. 28 was followed. Correct clones were identified by colony PCR followed by Sanger sequencing. Plasmids were injected into the attP1 site on the second chromosome (Bloomington *Drosophila* Stock Center [BDSC]: 8621) by Rainbow Transgenic (Rainbow transgenic flies Inc.). Individual flies were crossed to *w^1118^* (BDSC: 3605) and screened for red eye color. Red eyed flies were balanced using *w;CyO/Sco;MKRS/TM6B* (BDSC: 3703).

### FACS Sorting of *Drosophila* Neurons.

Neurons of interest were genetically labeled with *eGFP* using *nSyb*-GAL4 (for all neurons) or *clk856*-GAL4 (for clock neurons). About 2-wk-old male flies were entrained to the appropriate LD cycles. Brains of flies of the appropriate genotypes were dissected at ZT02 and ZT14 in ice-cold Schneider’s medium (SM) and stored on ice until all dissections were completed (about 10 for *nSyb* and 30 for *clk856*). Brains were incubated with an enzyme solution (SM with 0.75ug/ul collagenase and 0.04 μg/ul dispase) at room temperature (RT) for 30 mins and then washed with SM, and triturated in SM. The volume was then brought up to ∼1 mL and filtered through a 40-μm filter fitted on a FACS tube. GFP+ neurons of interest were isolated using the FACS Melody instrument (BD) with sorting gates set by comparing to a GFP-negative neuron sample. A total of 500 clock neurons or 1,000 nSyb neurons were collected per sample in 100 µL lysis buffer (Dynabeads mRNA direct kit) and frozen on dry ice immediately after collection. Two sets of neurons were collected from each dissociated sample.

### cDNA Synthesis and Library Preparation for Bulk Sequencing.

PolyA mRNA was isolated from the frozen cell samples using the Dynabeads mRNA direct kit (Thermo fisher 61011). Subsequently, complementary DNA (cDNA) was prepared using the method described in Picelli et al. (54). cDNA integrity and concentration were assessed using a High Sensitivity D5000 ScreenTape (Agilent 5067-5592). A total of ∼500 pg of cDNA was used as the input to make sequencing libraries with the Illumina Nextera XT DNA Library Preparation Kit (FC-131-1096) with nine PCR cycles. Final libraries were quantified on a High Sensitivity D1000 ScreenTape on the TapeStation (5067-5584).

Libraries were run on the Illumina NextSeq 550 sequencing system. Reads were aligned to the dm6 version of the *Drosophila* genome using STAR (55). PCR duplicates were removed using Picard Tools (Picard Toolkit 2019. Broad Institute, GitHub Repository, https://broadinstitute.github.io/picard/; Broad Institute). Differential expression analysis between *nSyb* neurons and clock neurons was performed using the Bioconductor package edgeR (33). Raw data were submitted to the Gene Expression Omnibus (GEO) repository and are available using the accession number GSE202407.

### scRNAseq Analysis.

For details on scRNAseq procedures, refer to Ma et al. (27). In order to identify differentially expressed GPCRs in clock neurons, we first computed all marker genes in each cluster using the *FindAllMarkers* function of the Seurat package. Using a negative binomial generalized linear model, the batch effect from sequencing depth and conditions was regressed out. We next used an adjusted *P* value significance of 0.05 and fold change cutoff of 1.25. GPCRs matching these criteria are regarded as differentially expressed in clock neurons. Their expression was plotted by the ComplexHeatmap package.

Annotated single-cell clustering data were used as the basis for GPCR-based reclustering using Seurat V4 in R (56). For the downstream analysis, only cells from the 17 high-confidence annotated clusters were used. GPCR-based reclustering was done by restricting the variable features to GPCRs by setting the features argument of the ScaleData Seurat function to all genes identified as GPCRs from Hanlon and Andrew (4) that were detectable in all timepoints. For the standard clustering, the FindVariableFeatures function with default settings was used to determine variable genes.

For both approaches, principal components (PCs) were determined using the RunPCA function, and the first 20 PCs were selected for clustering based on visual inspection of the ElbowPlots. Communities were generated using the standard workflow functions FindNeighbors, FindClusters, and RunUMAP with default settings. The resolution argument of the FindClusters argument was set at the default of 0.8 after experimentation with resolutions as low as 0.5. Higher values for resolution and increased numbers of PCs did not improve clustering results separately or in combination for the GPCR clustering.

The cell cluster identities published in Ma et al. (27) were stored in the Seurat metadata and used to assess the correspondence between the annotated clusters and the GPCR-based clustering. In general, the GPCR clusters correspond to a single annotated cluster and vice versa. There are two exceptions, as follows: the fusion of the two LNd clusters 9 and 12 into a single cluster and the fission of the DN1p cluster 4 into two similarly sized clusters. The split of the DN1p cluster is characterized by a nearly twofold difference in average levels of *FMRFaR*, which is expressed in subsets of DN1p neurons.

### Targeted Genomic Sequencing.

To analyze the potency of our guide library, we established a targeted genomic sequencing approach similar to the 16S metagenomic sequencing library preparation protocol (Illumina 15044223). In short, we generated three pairs of primers for each gene (*SI Appendix*, Table S3). Each pair of primers was designed to amplify 230- to 270-bp-long genomic regions centered around the predicted guide cut sites. To analyze the potency of our guide library to mutate genes in a cell-specific manner, we labeled the clock network with GFP (*clk856 > EGFP*) and expressed *Cas9* along with the guide of interest. We FACS sorted (see above) 2,000 GFP+ and 2,000 GFP− cells and extracted DNA using a DNA extraction buffer. We then used this extract and performed a PCR with all six primers for a gene of interest using an ExTaq polymerase. After Dynabeads mediated cleanup, adapters were ligated followed by another round of cleanup. Final libraries were quantified on a High Sensitivity D1000 ScreenTape on the TapeStation (5067-5584). Libraries were then pooled and sequenced using the miseq platform (Genewiz Inc.). Libraries were analyzed using Crispresso2 (57).

### Behavioral Analysis.

Two- to 7-d-old male flies were individually placed into glass tubes with food (2% agar, 4% sucrose) on one end and a plug to close the tube on the other end. These tubes were subsequently placed into *Drosophila* Activity Monitors (DAMs; Trikinetics Inc.), and a computer recorded the number of infrared light beam interruptions in 1-min intervals. Flies were recorded for 1 wk in LD 12:12 followed by DD for another week. Each experiment was performed at least twice.

To allow for proper entrainment to the LD cycle, we used the last 4 d of LD to analyze sleep, defined as 5 min of inactivity (58, 59). We then created average sleep profiles by averaging the amount of sleep across days and flies for each genotype in half-hour bins. To analyze sleep in more detail, we split the sleep amount into four sections of 6 h each, as follows: morning (ZT21 to ZT3), siesta (ZT3 and ZT9), evening (ZT9 and ZT15), and night (ZT15 to ZT21). Individual values were plotted as scatter plots, and statistical analysis was performed using a one-way ANOVA followed by a post hoc Tukey test (http://www.astatsa.com). *P* < 0.05 compared to all control groups was considered significant.

To analyze changes in rhythmicity, we performed a χ^2^ analysis for rhythmicity and analyzed the speed of the clock. Individual values were compared using a one-way ANOVA followed by a post hoc Tukey test (astatsa.com); *P* < 0.05 compared to all control groups was considered statistically significant.

### Immunohistochemistry.

Two- to 7-d-old male flies were fixed for 2 h 45min in 4% PFA in PBST (phosphate buffered saline including 0.5% TritonX). After rinsing 5× for 10 min each in PBST, brains were dissected and blocked for 2 h in 5% normal goat serum in PBST. Primary antibodies were applied overnight at RT. Primary antibodies were the following: chicken anti-GFP (1:1,500, abcam), rabbit anti-PER (1:1,000; 60), mouse anti-PDF (1:1,000, DSHB), and rat anti-RFP (1:500, chromotec). After rinsing 5× with PBST, secondary antibodies (1:200, Alexa Fluor, Fisher Scientific) were applied for 2 h at RT followed by rinsing 5× for 10 min each with PBST. Brains were subsequently mounted on glass slides using Vectashield mounting medium (Vector laboratories) and imaged using the Leica SP5 confocal microscope. For PER quantification we used a 3×3-pixel area to determine the staining intensity of neurons and corrected by measuring three different background intensities as previously described (61).

### Neuronal Tracing using *trans*-Tango.

The *trans*-Tango technique allows for anterograde transsynaptic tracing (43). UAS-*RFP*, *trans*Tango*; QUAS-GCaMP* flies were crossed to either *TH*-GAL4 (*SI Appendix*, Fig. S9) or *per-*AD *Vglut*-DBD split GAL4 (Fig. 5*B*) flies to identify the respective downstream targets. The upstream neurons (*TH*-GAL4 or *per*-AD *Vglut*-DBD) were labeled with RFP, whereas their synaptic downstream partners were labeled with GCaMP. Two- to 7-d-old male flies were stained using anti-GFP (to visualize GCaMP) and anti-period (to identify clock neurons) using the immunostaining protocol described above.

## Supplementary Material

Supplementary File

## Data Availability

RNAseq data have been deposited in GEO (GSE202407) (62).
