# Supplementary material for: Dopamine and GPCR-mediated modulation of DN1 clock neurons gates the circadian timing of sleep
Source: Proc Natl Acad Sci U S A. 2022 Aug 15;119(34):e2206066119. doi: 10.1073/pnas.2206066119 (PMC9407311; doi:10.1073/pnas.2206066119)
Supplement: Supplementary File [file pnas.2206066119.sapp.pdf]

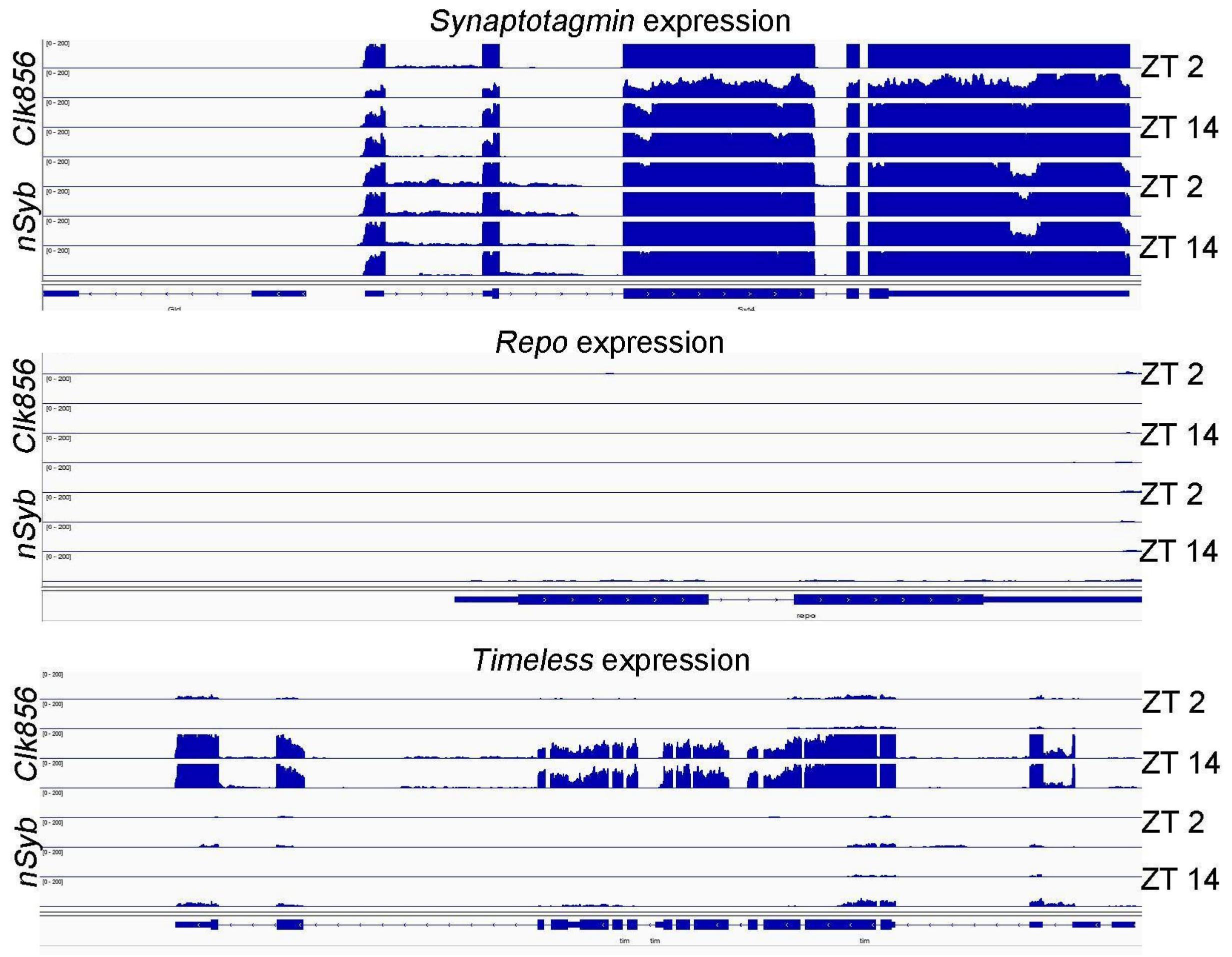

Figure S1: Bulk sequencing of FACS sorted neurons shows expected enrichment of key genes. Upper panel: Sorted clock neurons (*clk856>EGFP*) and sorted random neurons (*nSyb>EGFP*) show high expression of the neuronal marker *synaptotagmin*. Middle panel: Sorted clock neurons (*clk856>EGFP*) and sorted random neurons (*nSyb>EGFP*) show no expression of the glial marker *repo* indicating no contamination by glial cells. Lower Panel: Sorted clock neurons (*clk856>EGFP*) show high *timeless* expression at ZT14 and little expression at ZT2. Random neurons (*nSyb>EGFP*) show strongly reduced levels of *timeless* at all time points indicating successful enrichment of clock neurons. Maximum set to 200 reads for all graphs.

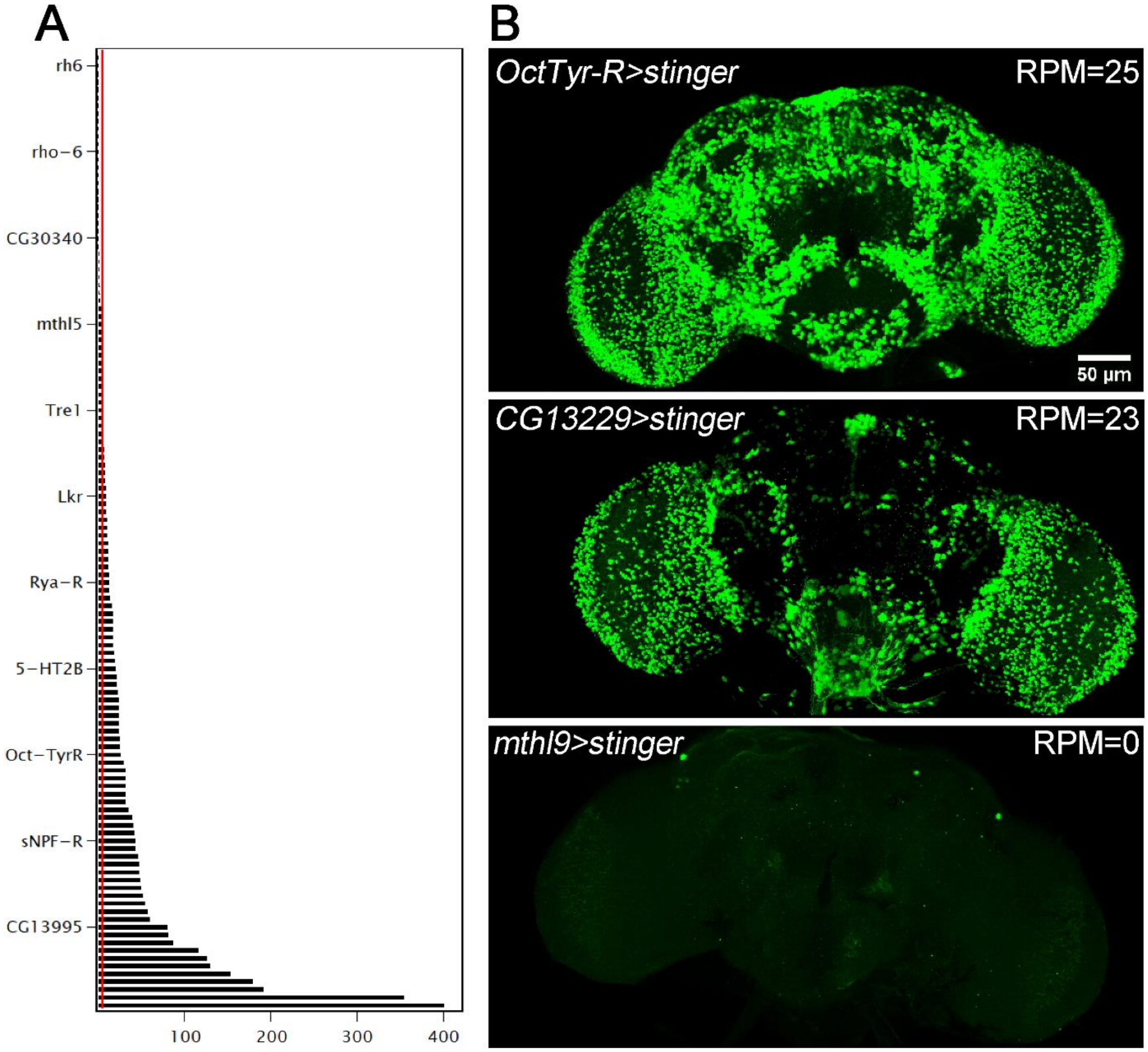

Figure S2: GPCRs are differentially expressed in the brain. A Normalized expression (RPM) of GPCRs from isolated neurons (*nSyb>GFP*). Expression of GPCRs varies strongly from gene to gene. **B** Whole mount brains of flies expressing nuclear GFP under the control of specific GPCRs. From top to bottom: *Oct-Tyr-R>stinger*, *CG13229>stinger* and *mthl9>stinger*. The number of nuclei labeled with GFP corresponds to the number of normalized reads as indicated in the top right corner of each image.

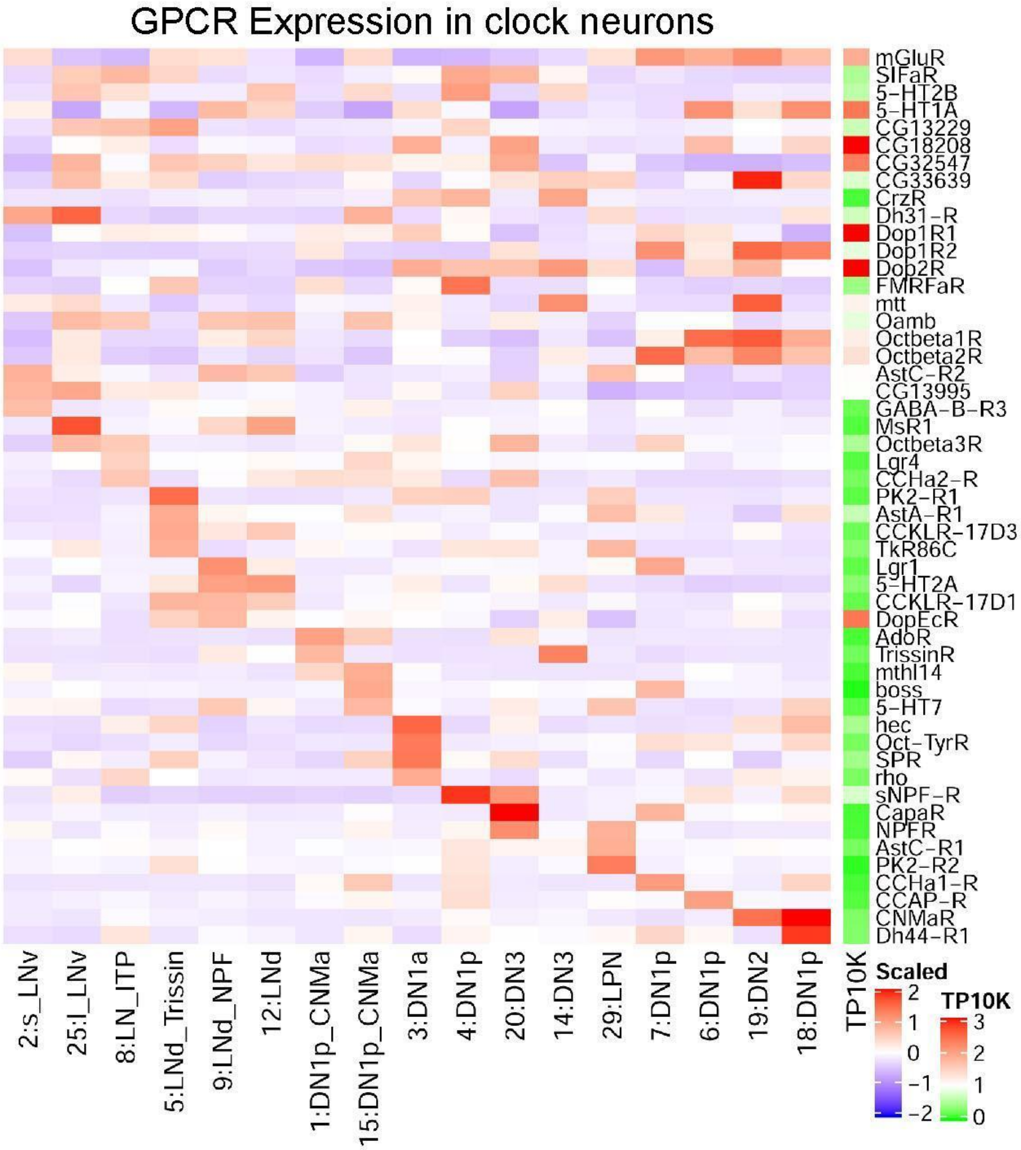

Figure S3: GPCRs are highly differentially expressed in the clock neuron network based on single-cell RNA sequencing. Heatmap on the right (green: low expression red: high expression) indicates the overall expression level of the specific GPCR. Heatmap on the left (blue: low expression red: high expression) represents the expression levels of specific GPCRs within the 17 high confidence clock neuron clusters.

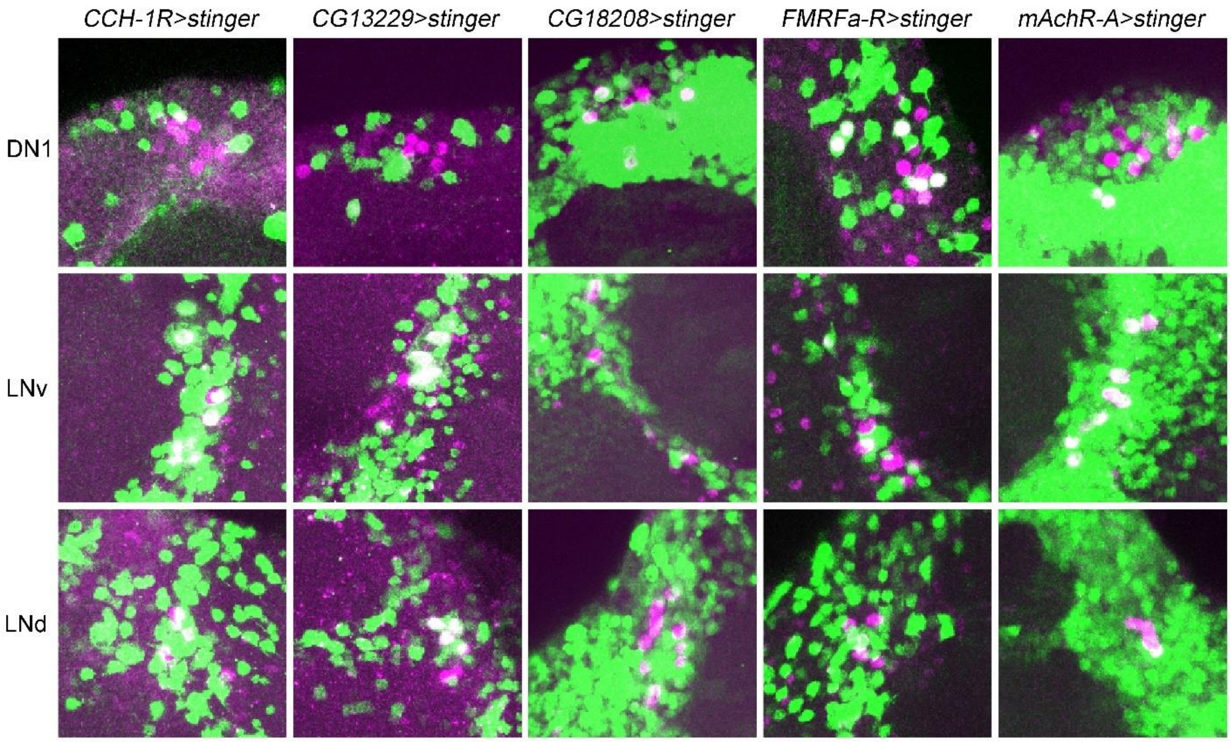

Figure S4: GPCRs are differentially expressed based on endogenous GAL4 expression. Whole mount brains were labeled with anti-GFP (green) and anti-PER (magenta). To analyze colocalization, we generated z-stacks of all the stacks containing the cells of interest. From top to bottom: DN1ps (posterior brain), ventro-lateral neurons (PDF-cells) and LNds. Genotypes are indicated on top of each row.

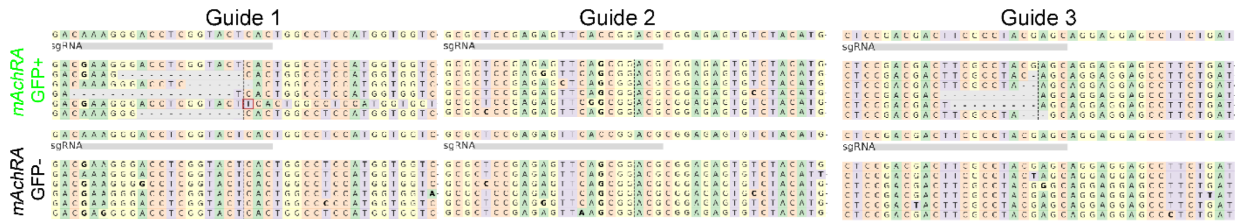

Figure S5: Guide library efficiently mutates GPCRs of choice in a cell-type specific manner. Mutagenesis of *mAchR-A* in clock neurons. Guides 1 and 3 cause deletions in GFP positive neurons, whereas no deletions were detected in Guide 2 and GFP negative cells.

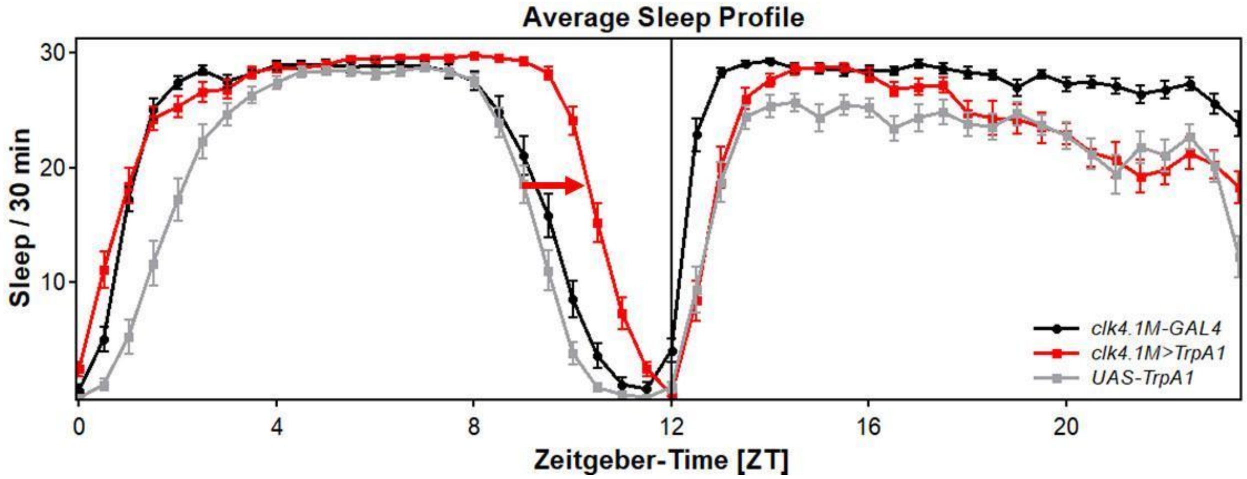

Figure S6: Activating DN1 neurons increases the siesta. Mild activation (26°C) of DN1ps using the temperature sensitive cation channel *TrpA1* increases sleep in the second half of the day (red line) compared to both controls (grey and black lines), whereas there were no major effects on sleep at any other time of the day.

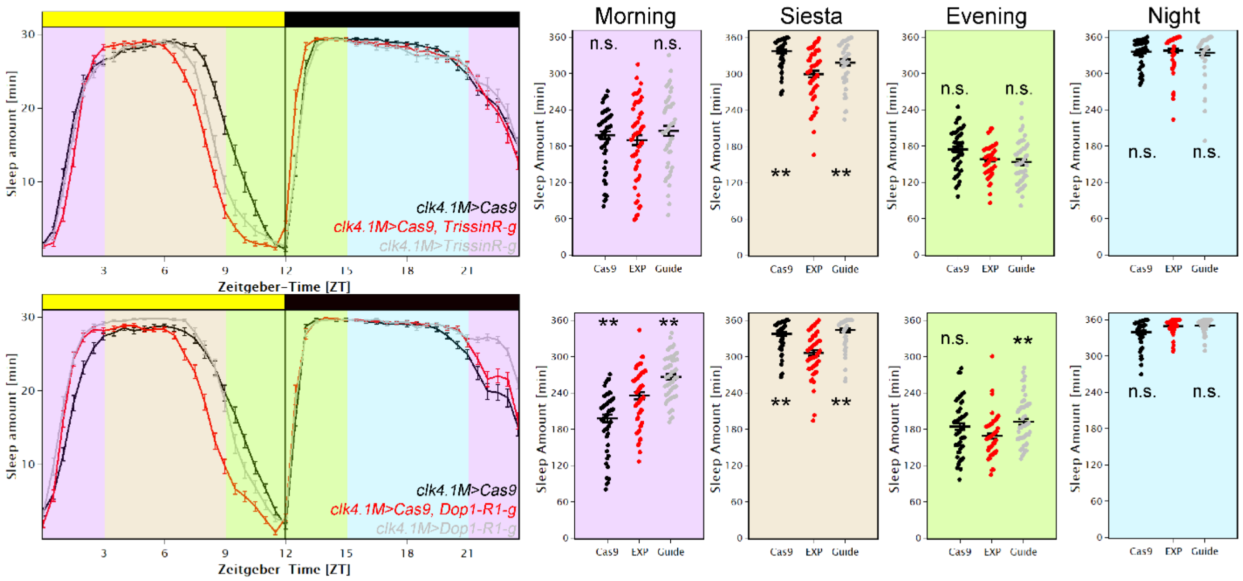

Figure S7: Mutagenesis of *Dop1R1* and *TrissinR* in DN1ps affects siesta sleep Upper panel: Average sleep profile of male flies in which *TrissinR* was mutated in the DN1ps (red) and controls (grey and black). Error bars represent SEM. Background colors indicate 4x6h periods which were quantified. Quantification of sleep is separated into 4 different time zones: Morning (ZT21-ZT3), Siesta (ZT3-ZT9), Evening (ZT9-ZT15) and Night (ZT15-ZT21). Removing *TrissinR* in the DN1ps significantly reduced sleep during the siesta ($p<0.01$), whereas other times of day were not affected. Lower panel: Average sleep profile of male flies in which *Dop1R1* was mutated in the DN1ps (red) and controls (grey and black). Error bars represent SEM. Background colors indicate 4x6h periods which were quantified. Quantification of sleep is separated into 4 different time zones: Morning (ZT21-ZT3), Siesta (ZT3-ZT9), Evening (ZT9-ZT15) and Night (ZT15-ZT21). Removing *Dop1R1* in the DN1ps significantly reduced sleep during the siesta ($p<0.01$), whereas other times of day were not affected.

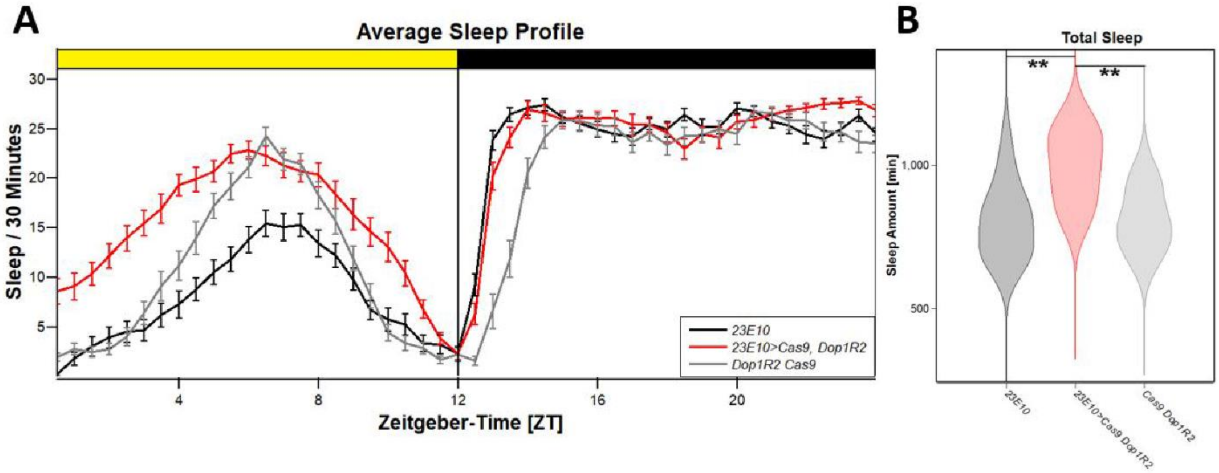

Figure S8: Dop1R2 reduces sleep in the dorsal fan shaped body. A Average sleep profile of female flies with mutated *Dop1R2* in the dFb (red) and controls (grey and black). Error bars represent SEM. Experimental flies show increased sleep especially during daytime. **B** Quantification of total sleep. Experimental flies show significantly increased sleep amounts compared to both controls ($p<0.01$).

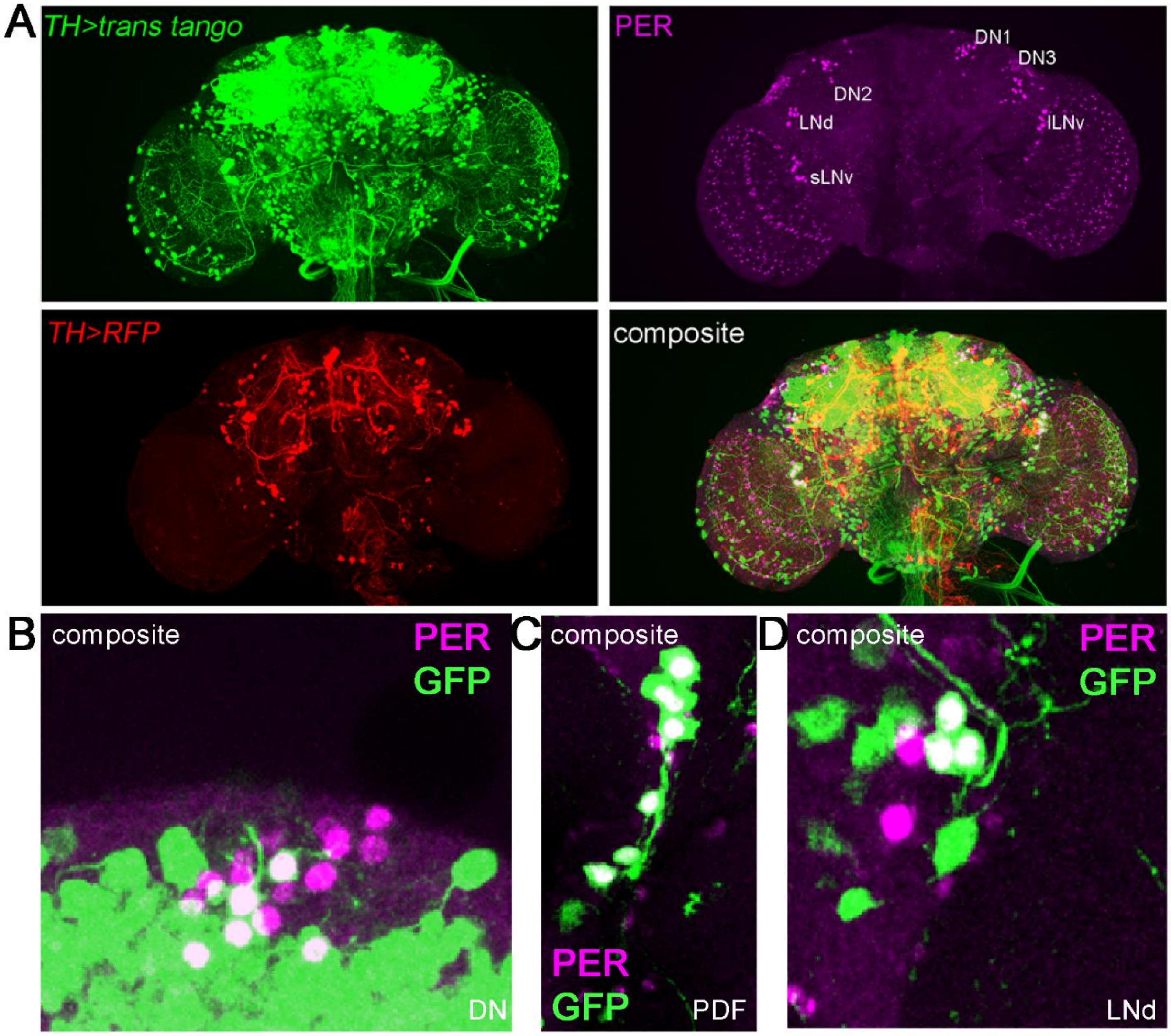

Figure S9: Dopaminergic neurons connect to a variety of clock neurons. A Whole mount brain of *TH>RFP*, *trans-tango; QUAS-Gcamp* stained with anti-GFP (green, labeling downstream targets of TH neurons), anti-RFP (red, labels TH neurons) and anti-PER (magenta, labeling clock cells). The TH neurons connect to many cells in the fly brain. **B** TH neurons connect to approximately 4 DN1ps and DN2s. **C** TH neurons connect to sLNvs and lLNvs. **D** TH neurons connect to 3 LNds.

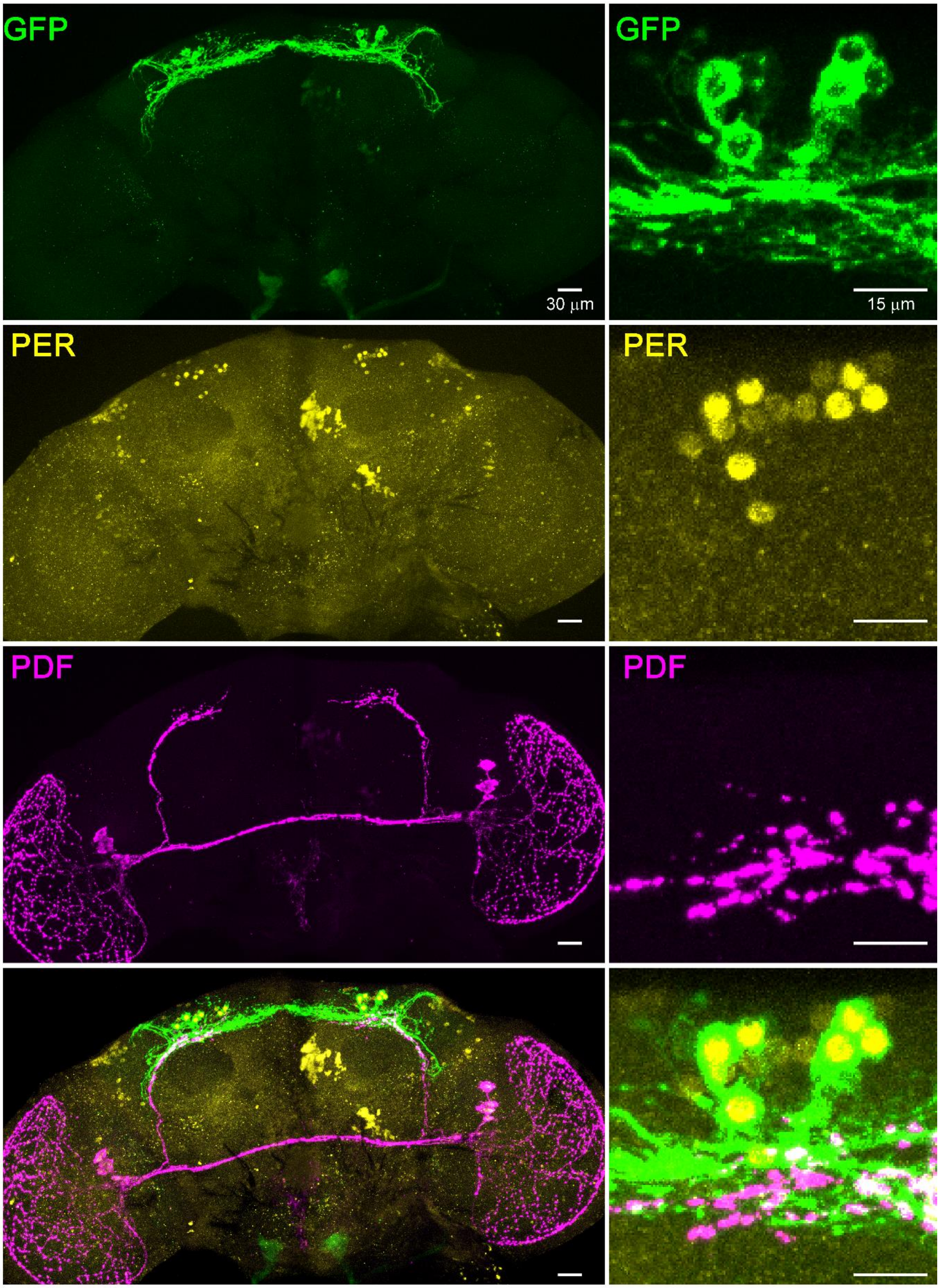

Figure S10: *per-AD Vglut-DBD>EGFP* expressing flies have 7-8 GFP+ DN1ps. Brains stained against anti-GFP (green), anti-PER (yellow) and anti-PDF (magenta).

Table S1: Flies used in this study

Genotype	Description	Reference / Source
clk856-GAL4	Expresses GAL4 in most clock neurons	(1)
UAS-EGFP	Expresses EGFP under UAS control	BDSC 5430
nSyb-GAL4	Expresses GAL4 in neurons	BDSC 51635
UAS-stinger	Expresses nuclear GFP under UAS control	BDSC 84278
Dh31-R-Gal4-RA/C	DKI0062	(2)
sNPF-R-Gal4	WCKI1115	(2)
Oct-TyrR-GAL4-T1/Sb		(2)
CG13229-Gal4	DKI0133	(2)
mthl9-Gal4	WCKI1036	(2)
FMRFaR-Gal4	WCKI1117	(2)
CG18208-Gal4	WCKI1100	(2)
CCHa1-R-Gal4	DKI0077	(2)
mAChR-A-Gal4	DKI0218	(2)
UAS-Cas9.P2 (attP40)	Expresses Cas9 under UAS control	BDSC 58985
clk4.1M-GAL4	Expresses GAL4 in DN1s	(3)
UAS-TrpA1		BDSC 26263
23E10-GAL4		BDSC 49032
per-AD		BDSC 70551
Vglut-DBD		BDSC 69036
UAS-trans-tango UAS-RFP QUAS-Gcamp		Modified from BDSC 77124
UAS-5-HT1A-g	UAS-guide-RNA line	This study
UAS-5HT1B-g	UAS-guide-RNA line	This study
UAS-5HT2A-g	UAS-guide-RNA line	This study
UAS-5HT2B-g	UAS-guide-RNA line	This study
UAS-5HT7-g	UAS-guide-RNA line	This study
UAS-AdoR-g	UAS-guide-RNA line	This study
UAS-AkhR-g	UAS-guide-RNA line	This study
UAS-AstA-R1-g	UAS-guide-RNA line	This study

UAS-AstA-R2-g	UAS-guide-RNA line	This study
UAS-AstC-R1-g	UAS-guide-RNA line	This study
UAS-AstC-R2-g	UAS-guide-RNA line	This study
UAS-boss-g	UAS-guide-RNA line	This study
UAS-CapaR-g	UAS-guide-RNA line	This study
UAS-CCAP-R-g	UAS-guide-RNA line	This study
UAS-CCHa1-R-g	UAS-guide-RNA line	This study
UAS-CCHa2-R-g	UAS-guide-RNA line	This study
UAS-CCKLR-14D1-g	UAS-guide-RNA line	This study
UAS-CCKLR-17D3-g	UAS-guide-RNA line	This study
UAS-CG11318-g	UAS-guide-RNA line	This study
UAS-CG12290-g	UAS-guide-RNA line	This study
UAS-CG12796-g	UAS-guide-RNA line	This study
UAS-CG13229-g	UAS-guide-RNA line	This study
UAS-CG13575-g	UAS-guide-RNA line	This study
UAS-CG13579-g	UAS-guide-RNA line	This study
UAS-CG13995-g	UAS-guide-RNA line	This study
UAS-CG15556-g	UAS-guide-RNA line	This study
UAS-CG15614-g	UAS-guide-RNA line	This study
UAS-CG15744-g	UAS-guide-RNA line	This study
UAS-CG18208-g	UAS-guide-RNA line	This study
UAS-CG30340-g	UAS-guide-RNA line	This study
UAS-CG31760-g	UAS-guide-RNA line	This study
UAS-CG32447-g	UAS-guide-RNA line	This study
UAS-CG32547-g	UAS-guide-RNA line	This study
UAS-CG33639-g	UAS-guide-RNA line	This study
UAS-CG4313-g	UAS-guide-RNA line	This study
UAS-CG44153-g	UAS-guide-RNA line	This study
UAS-CG7497-g	UAS-guide-RNA line	This study
UAS-Cirl-g	UAS-guide-RNA line	This study
UAS-CNMaR-g	UAS-guide-RNA line	This study
UAS-CrzR-g	UAS-guide-RNA line	This study

UAS-Dh31-R-g	UAS-guide-RNA line	This study
UAS-Dh44-R1-g	UAS-guide-RNA line	This study
UAS-Dh44-R2-g	UAS-guide-RNA line	This study
UAS-Dop1R1-g	UAS-guide-RNA line	This study
UAS-Dop1R2-g	UAS-guide-RNA line	This study
UAS-Dop2R-g	UAS-guide-RNA line	This study
UAS-DopEcR-g	UAS-guide-RNA line	This study
UAS-ETHR-g	UAS-guide-RNA line	This study
UAS-FMRFaR-g	UAS-guide-RNA line	This study
UAS-fz-g	UAS-guide-RNA line	This study
UAS-fz2-g	UAS-guide-RNA line	This study
UAS-fz3-g	UAS-guide-RNA line	This study
UAS-fz4-g	UAS-guide-RNA line	This study
UAS-GABA-B-R1-g	UAS-guide-RNA line	This study
UAS-GABA-B-R2-g	UAS-guide-RNA line	This study
UAS-GABA-B-R3-g	UAS-guide-RNA line	This study
UAS-hec-g	UAS-guide-RNA line	This study
UAS-Lgr1-g	UAS-guide-RNA line	This study
UAS-Lgr3-g	UAS-guide-RNA line	This study
UAS-Lgr4-g	UAS-guide-RNA line	This study
UAS-Lkr-g	UAS-guide-RNA line	This study
UAS-mAchR-A-g	UAS-guide-RNA line	This study
UAS-mAchR-B-g	UAS-guide-RNA line	This study
UAS-mAchR-C-g	UAS-guide-RNA line	This study
UAS-mGluR-g	UAS-guide-RNA line	This study
UAS-moody-g	UAS-guide-RNA line	This study
UAS-MsR1-g	UAS-guide-RNA line	This study
UAS-MsR2-g	UAS-guide-RNA line	This study
UAS-mth-g	UAS-guide-RNA line	This study
UAS-mthl1-g	UAS-guide-RNA line	This study
UAS-mthl10-g	UAS-guide-RNA line	This study
UAS-mthl11-g	UAS-guide-RNA line	This study

UAS-mthl12-g	UAS-guide-RNA line	This study
UAS-mthl13-g	UAS-guide-RNA line	This study
UAS-mthl14-g	UAS-guide-RNA line	This study
UAS-mthl15-g	UAS-guide-RNA line	This study
UAS-mthl2-g	UAS-guide-RNA line	This study
UAS-mthl3-g	UAS-guide-RNA line	This study
UAS-mthl4-g	UAS-guide-RNA line	This study
UAS-mthl5-g	UAS-guide-RNA line	This study
UAS-mthl6-g	UAS-guide-RNA line	This study
UAS-mthl7-g	UAS-guide-RNA line	This study
UAS-mthl8-g	UAS-guide-RNA line	This study
UAS-mthl9-g	UAS-guide-RNA line	This study
UAS-mtt-g	UAS-guide-RNA line	This study
UAS-ninaE-g	UAS-guide-RNA line	This study
UAS-NPFR-g	UAS-guide-RNA line	This study
UAS-Oamb-g	UAS-guide-RNA line	This study
UAS-Octbeta1R-g	UAS-guide-RNA line	This study
UAS-Octbeta2R-g	UAS-guide-RNA line	This study
UAS-Octbeta3R-g	UAS-guide-RNA line	This study
UAS-Oct-TyrR-g	UAS-guide-RNA line	This study
UAS-PDFR-g	UAS-guide-RNA line	This study
UAS-PK1-R-g	UAS-guide-RNA line	This study
UAS-P2-R1-g	UAS-guide-RNA line	This study
UAS-P2-R2-g	UAS-guide-RNA line	This study
UAS-Proc-R-g	UAS-guide-RNA line	This study
UAS-rh2-g	UAS-guide-RNA line	This study
UAS-rh3-g	UAS-guide-RNA line	This study
UAS-rh4-g	UAS-guide-RNA line	This study
UAS-rh5-g	UAS-guide-RNA line	This study
UAS-rh6-g	UAS-guide-RNA line	This study
UAS-rh7-g	UAS-guide-RNA line	This study
UAS-rho-g	UAS-guide-RNA line	This study

UAS-rho-4-g	UAS-guide-RNA line	This study
UAS-rho-5-g	UAS-guide-RNA line	This study
UAS-rho-6-g	UAS-guide-RNA line	This study
UAS-rho-7-g	UAS-guide-RNA line	This study
UAS-rk-g	UAS-guide-RNA line	This study
UAS-ru-g	UAS-guide-RNA line	This study
UAS-Rya-R-g	UAS-guide-RNA line	This study
UAS-smo-g	UAS-guide-RNA line	This study
UAS-smog-g	UAS-guide-RNA line	This study
UAS-sNPF-R-g	UAS-guide-RNA line	This study
UAS-SPR-g	UAS-guide-RNA line	This study
UAS-stan-g	UAS-guide-RNA line	This study
UAS-stet-g	UAS-guide-RNA line	This study
UAS-TkR86C-g	UAS-guide-RNA line	This study
UAS-TkR99D-g	UAS-guide-RNA line	This study
UAS-Tre1-g	UAS-guide-RNA line	This study
UAS-TrissinR-g	UAS-guide-RNA line	This study
UAS-TyrR	UAS-guide-RNA line	This study
UAS-TyRII	UAS-guide-RNA line	This study

Table S2: Primers used to generate UAS-guide lines.

Gene name	#	Sequence
CrzR	1	GGCCCGGGTTCGATTCCCGGCCGATGCAAACATGATCGACGTGGGTGTGTTTCAGAGCTATGCTGGAAA
CrzR	2	GCGTGAAGGTGGCGTACATCTGCACCAGCCGGGAATCGAACC
CrzR	3	GATGTACGCCACCTTCACGCGTTTCAGAGCTATGCTGGAAAC
CrzR	4	TTTAACTTGCTATTTCTAGCTCTAAAACCGCCGCTCGACTTGCCCCCTTGCACCAGCCGGGAATCGAAC
AkhR	1	GGCCCGGGTTCGATTCCCGGCCGATGCAAAGGATATGGTCTTCAATGAGTTTCAGAGCTATGCTGGAAA
AkhR	2	TCACGTAGCTGGACAGATACTGCACCAGCCGGGAATCGAACC
AkhR	3	GTATCTGTCCAGCTACGTGAGTTTCAGAGCTATGCTGGAAAC
AkhR	4	TTTAACTTGCTATTTCTAGCTCTAAAACCGAATGTTGTAGAGTCCGTATGCACCAGCCGGGAATCGAAC
CCAP-R	1	GGCCCGGGTTCGATTCCCGGCCGATGCAAGTCACGACTTGGAGACGGGGTTTCAGAGCTATGCTGGAAA
CCAP-R	2	AATCGTCTTTACGATGATCGTGCACCAGCCGGGAATCGAACC
CCAP-R	3	CGATCATCGTAAAGACGATTGTTTCAGAGCTATGCTGGAAAC
CCAP-R	4	TTTAACTTGCTATTTCTAGCTCTAAAACACGACCAACCGTGTGGCAGCTGCACCAGCCGGGAATCGAAC
CG30340	1	GGCCCGGGTTCGATTCCCGGCCGATGCAGGGATGCGTGGGCTGCAAGCGTTTCAGAGCTATGCTGGAAA
CG30340	2	GGTGATCTCCCCATTGGGCTTGCACCAGCCGGGAATCGAACC
CG30340	3	AGCCCAATGGGGAGATCACCGTTTCAGAGCTATGCTGGAAAC
CG30340	4	TTTAACTTGCTATTTCTAGCTCTAAAACTTCTTCATAAAGGCACAGTATGCACCAGCCGGGAATCGAAC
CG13995	1	GGCCCGGGTTCGATTCCCGGCCGATGCAAGTGGTGGGAGAACTCGTATGTTTCAGAGCTATGCTGGAAA
CG13995	2	ATCGCGGATGCTTTACTGTGTGCACCAGCCGGGAATCGAACC
CG13995	3	CACAGTAAAGCATCCGCGATGTTTCAGAGCTATGCTGGAAAC
CG13995	4	TTTAACTTGCTATTTCTAGCTCTAAAACGCCTGCTCGGTTGATGCGCTTGCACCAGCCGGGAATCGAAC
CG4313	1	GGCCCGGGTTCGATTCCCGGCCGATGCATTTCCATAATGATACCCACCGTTTCAGAGCTATGCTGGAAA
CG4313	2	CTGGACGTGGTCACCTTCTCTGCACCAGCCGGGAATCGAACC
CG4313	3	GAGAAGGTGACCACGTCCAGGTTTCAGAGCTATGCTGGAAAC
CG4313	4	TTTAACTTGCTATTTCTAGCTCTAAAACTGTGAAGGAGGTGGTGCGCATGCACCAGCCGGGAATCGAAC
moody	1	GGCCCGGGTTCGATTCCCGGCCGATGCAGTTCCCCCGGCGGACGCGACGTTTCAGAGCTATGCTGGAAA
moody	2	CCACGTTGCGCACCTTGGGATGCACCAGCCGGGAATCGAACC
moody	3	TCCCAAGGTGCGCAACGTGGGTTTCAGAGCTATGCTGGAAAC
moody	4	TTTAACTTGCTATTTCTAGCTCTAAAACTTGGTCTTCCCGAAGGGCAGTGCACCAGCCGGGAATCGAAC
Tre1	1	GGCCCGGGTTCGATTCCCGGCCGATGCAGCCTGTGTCTTTGTGACGATGTTTCAGAGCTATGCTGGAAA
Tre1	2	ACCATGCTGAGGAGTGAAACTGCACCAGCCGGGAATCGAACC

Tre1	3	GTTTCACTCCTCAGCATGGTGTTTCAGAGCTATGCTGGAAAC
Tre1	4	TTTAACTTGCTATTTCTAGCTCTAAAACTCATGACGGTCAGTCGATTGTGCACCAGCCGGGAATCGAAC
CG15614	1	GGCCCGGGTTCGATTCCCGGCCGATGCAGTTGTGCTCTTTGTGCTCTAGTTTCAGAGCTATGCTGGAAA
CG15614	2	CAACAATGATGCAGCGAGCCTGCACCAGCCGGGAATCGAACC
CG15614	3	GGCTCGCTGCATCATTGTTGGTTTCAGAGCTATGCTGGAAAC
CG15614	4	TTTAACTTGCTATTTCTAGCTCTAAAACATGACCAGAAATATGGCTGGGTGCACCAGCCGGGAATCGAAC
CG7497	1	GGCCCGGGTTCGATTCCCGGCCGATGCAGATTATTGGTGTTATTATCAGTTTCAGAGCTATGCTGGAAA
CG7497	2	GTAGAGCCACCAGATTGTTGTGCACCAGCCGGGAATCGAACC
CG7497	3	CAACAATCTGGTGGCTCTACGTTTCAGAGCTATGCTGGAAAC
CG7497	4	TTTAACTTGCTATTTCTAGCTCTAAAACATCACACAGAGCAAGGTGTGTGCACCAGCCGGGAATCGAAC
FMRFaR	1	GGCCCGGGTTCGATTCCCGGCCGATGCATGAGTGGTACAGCGGTTGCGGTTTCAGAGCTATGCTGGAAA
FMRFaR	2	ATAGGGAATACCGCCGGCGATGCACCAGCCGGGAATCGAACC
FMRFaR	3	TCGCCGGCGGTATTCCCTATGTTTCAGAGCTATGCTGGAAAC
FMRFaR	4	TTTAACTTGCTATTTCTAGCTCTAAAACGCCTGTATATCAGACAATTTTGCACCAGCCGGGAATCGAAC
CNMaR	1	GGCCCGGGTTCGATTCCCGGCCGATGCAGAGTATATAACTAGCAGTAGGTTTCAGAGCTATGCTGGAAA
CNMaR	2	GATGAGTTCGACGAGTTCGATGCACCAGCCGGGAATCGAACC
CNMaR	3	TCGAACTCGTCGAACTCATCGTTTCAGAGCTATGCTGGAAAC
CNMaR	4	TTTAACTTGCTATTTCTAGCTCTAAAACCCCGTTGAGCGGATGAAACCTGCACCAGCCGGGAATCGAAC
Proc-R	1	GGCCCGGGTTCGATTCCCGGCCGATGCAAGTATCATTTCAAGCTCTACGTTTCAGAGCTATGCTGGAAA
Proc-R	2	ATCGTAATTGTATGCTCGAATGCACCAGCCGGGAATCGAACC
Proc-R	3	TTCGAGCATACAATTACGATGTTTCAGAGCTATGCTGGAAAC
Proc-R	4	TTTAACTTGCTATTTCTAGCTCTAAAACGTGACGGCGATGTCGTGGAATGCACCAGCCGGGAATCGAAC
CG33639	1	GGCCCGGGTTCGATTCCCGGCCGATGCAACCTGAGGGACGACTTCTATGTTTCAGAGCTATGCTGGAAA
CG33639	2	GATAAAGCTCCAGGTGGGCGTGCACCAGCCGGGAATCGAACC
CG33639	3	CGCCCACCTGGAGCTTTATCGTTTCAGAGCTATGCTGGAAAC
CG33639	4	TTTAACTTGCTATTTCTAGCTCTAAAACGTAGACCATCATGATGCGCATGCACCAGCCGGGAATCGAAC
SPR	1	GGCCCGGGTTCGATTCCCGGCCGATGCACGTACTGTACCAGTACCGCTGTTTCAGAGCTATGCTGGAAA
SPR	2	AACGGAGATGGTGTGGCACATGCACCAGCCGGGAATCGAACC
SPR	3	TGTGCCACACCATCTCCGTTGTTTCAGAGCTATGCTGGAAAC
SPR	4	TTTAACTTGCTATTTCTAGCTCTAAAACCGTTCGGGCCATGGGGGCGTTGCACCAGCCGGGAATCGAAC
CG13229	1	GGCCCGGGTTCGATTCCCGGCCGATGCAAGTGCCAGGGCTATTGCCAGGTTTCAGAGCTATGCTGGAAA
CG13229	2	GCGTGACAGTCAATCCGATGTGCACCAGCCGGGAATCGAACC

CG13229	3	CATCGGATTGACTGTCACGCGTTTCAGAGCTATGCTGGAAAC
CG13229	4	TTTAACTTGCTATTTCTAGCTCTAAAACCACGGCAGAAGTTTGATGAGTGCACCAGCCGGGAATCGAAC
MsR2	1	GGCCCGGGTTCGATTCCCGGCCGATGCAATGTCGCAGCCGCATTATTGGTTTCAGAGCTATGCTGGAAA
MsR2	2	AGGATACAGACAATCAGCGATGCACCAGCCGGGAATCGAACC
MsR2	3	TCGCTGATTGTCTGTATCCTGTTTCAGAGCTATGCTGGAAAC
MsR2	4	TTTAACTTGCTATTTCTAGCTCTAAAACACAGAGCACCGATGAGCCGCTGCACCAGCCGGGAATCGAAC
MsR1	1	GGCCCGGGTTCGATTCCCGGCCGATGCAGAAACTGAGCCGCTCTACTGGTTTCAGAGCTATGCTGGAAA
MsR1	2	GGTCGGCCACGGCCAGACCCTGCACCAGCCGGGAATCGAACC
MsR1	3	GGGTCTGGCCGTGGCCGACCGTTTCAGAGCTATGCTGGAAAC
MsR1	4	TTTAACTTGCTATTTCTAGCTCTAAAACGTAAGGCCAGGATCAATCGATGCACCAGCCGGGAATCGAAC
AstC-R2	1	GGCCCGGGTTCGATTCCCGGCCGATGCAACCAACGGCTGTGCCCATTCGTTTCAGAGCTATGCTGGAAA
AstC-R2	2	CCGACATGATCAACAGGAAGTGCACCAGCCGGGAATCGAACC
AstC-R2	3	CTTCCTGTTGATCATGTCGGGTTTCAGAGCTATGCTGGAAAC
AstC-R2	4	TTTAACTTGCTATTTCTAGCTCTAAAACTCGGAGCATTGTTGTTTCTCTGCACCAGCCGGGAATCGAAC
AstC-R1	1	GGCCCGGGTTCGATTCCCGGCCGATGCAGAACGAGAGCTTATATACCAGTTTCAGAGCTATGCTGGAAA
AstC-R1	2	GGCAATCGCTGAGACCACTTTGCACCAGCCGGGAATCGAACC
AstC-R1	3	AAGTGGTCTCAGCGATTGCCGTTTCAGAGCTATGCTGGAAAC
AstC-R1	4	TTTAACTTGCTATTTCTAGCTCTAAAACGCTTATTCATACAGGTAAAGTGCACCAGCCGGGAATCGAAC
AstA-R2	1	GGCCCGGGTTCGATTCCCGGCCGATGCAGGGATTCTTCGGCAACCTGCGTTTCAGAGCTATGCTGGAAA
AstA-R2	2	CATGATCATGCGCATGTAGATGCACCAGCCGGGAATCGAACC
AstA-R2	3	TCTACATGCGCATGATCATGGTTTCAGAGCTATGCTGGAAAC
AstA-R2	4	TTTAACTTGCTATTTCTAGCTCTAAAACGAAATTCTCGGAGAGGAAGGTGCACCAGCCGGGAATCGAAC
AstA-R1	1	GGCCCGGGTTCGATTCCCGGCCGATGCAAGCTGGCCAAAAGCCTCTTGGTTTCAGAGCTATGCTGGAAA
AstA-R1	2	AGACGGCCAGGTTGATTATCTGCACCAGCCGGGAATCGAACC
AstA-R1	3	GATAATCAACCTGGCCGTCTGTTTCAGAGCTATGCTGGAAAC
AstA-R1	4	TTTAACTTGCTATTTCTAGCTCTAAAACTGGTACTGATAAATCCTCACTGCACCAGCCGGGAATCGAAC
CCHa2-R	1	GGCCCGGGTTCGATTCCCGGCCGATGCATGTGCCCGTGCTGGACCGCCGTTTCAGAGCTATGCTGGAAA
CCHa2-R	2	GCTTTCCTGCGTGTAGACAATGCACCAGCCGGGAATCGAACC
CCHa2-R	3	TTGTCTACACGCAGGAAAGCGTTTCAGAGCTATGCTGGAAAC
CCHa2-R	4	TTTAACTTGCTATTTCTAGCTCTAAAACCTTGACGCAGATGCAGCAGATGCACCAGCCGGGAATCGAAC
CCHa1-R	1	GGCCCGGGTTCGATTCCCGGCCGATGCAGAGACACCCTACGTGCCCTAGTTTCAGAGCTATGCTGGAAA
CCHa1-R	2	ATCGCCGGATAATGCAGTCATGCACCAGCCGGGAATCGAACC

CCHa1-R	3	TGACTGCATTATCCGGCGATGTTTCAGAGCTATGCTGGAAAC
CCHa1-R	4	TTTAACTTGCTATTTCTAGCTCTAAAACCTCACGAAGTATAGGGCCACTGCACCAGCCGGGAATCGAAC
TrissinR	1	GGCCCGGGTTCGATTCCCGGCCGATGCATAATGACGACGGCCACCACAGTTTCAGAGCTATGCTGGAAA
TrissinR	2	GGCAGGATGGCTGCTGCGGCTGCACCAGCCGGGAATCGAACC
TrissinR	3	GCCGCAGCAGCCATCCTGCCGTTTCAGAGCTATGCTGGAAAC
TrissinR	4	TTTAACTTGCTATTTCTAGCTCTAAAACGCCCGCTGGTGATGGGCGTGTGCACCAGCCGGGAATCGAAC
PK2-R2	1	GGCCCGGGTTCGATTCCCGGCCGATGCACAACCTGACCAGCCTTCTCCGTTTCAGAGCTATGCTGGAAA
PK2-R2	2	TTTCCGAGAGAACGCTCTCCTGCACCAGCCGGGAATCGAACC
PK2-R2	3	GGAGAGCGTTCTCTCGGAAAGTTTCAGAGCTATGCTGGAAAC
PK2-R2	4	TTTAACTTGCTATTTCTAGCTCTAAAACGATGACTCGCGTTTGGGCGCTGCACCAGCCGGGAATCGAAC
PK2-R1	1	GGCCCGGGTTCGATTCCCGGCCGATGCACCCAATCAGAGTCCCTCCATGTTTCAGAGCTATGCTGGAAA
PK2-R1	2	TCGTTCTGGTAGACCACCGATGCACCAGCCGGGAATCGAACC
PK2-R1	3	TCGGTGGTCTACCAGAACGAGTTTCAGAGCTATGCTGGAAAC
PK2-R1	4	TTTAACTTGCTATTTCTAGCTCTAAAACGACCAAACTGTCGCGTCAGATGCACCAGCCGGGAATCGAAC
PK1-R	1	GGCCCGGGTTCGATTCCCGGCCGATGCACCAAGTACCCGTACGTGTTTGTTTCAGAGCTATGCTGGAAA
PK1-R	2	CCCGTGCAGGGGCGTAGATGTGCACCAGCCGGGAATCGAACC
PK1-R	3	CATCTACGCCCCTGCACGGGGTTTCAGAGCTATGCTGGAAAC
PK1-R	4	TTTAACTTGCTATTTCTAGCTCTAAAACTCCTCAGGCGGCGCGATTCGTGCACCAGCCGGGAATCGAAC
CapaR	1	GGCCCGGGTTCGATTCCCGGCCGATGCAGGAGAGGAGGACTATGCCTGGTTTCAGAGCTATGCTGGAAA
CapaR	2	GTTATAAATATGCCACCAAATGCACCAGCCGGGAATCGAACC
CapaR	3	TTTGGTGGCATATTTATAACGTTTCAGAGCTATGCTGGAAAC
CapaR	4	TTTAACTTGCTATTTCTAGCTCTAAAACAAGAGATCCGGATACTGATGTGCACCAGCCGGGAATCGAAC
ETHR	1	GGCCCGGGTTCGATTCCCGGCCGATGCAATCATGCTGCTGGGTGTGGTGTTTCAGAGCTATGCTGGAAA
ETHR	2	TCGTCTTCACAATCACGATGTGCACCAGCCGGGAATCGAACC
ETHR	3	CATCGTGATTGTGAAGACGAGTTTCAGAGCTATGCTGGAAAC
ETHR	4	TTTAACTTGCTATTTCTAGCTCTAAAACTGGTGCAGACGTAGCCGGCCTGCACCAGCCGGGAATCGAAC
TkR99D	1	GGCCCGGGTTCGATTCCCGGCCGATGCAAGCAACTGGAGCACCCCCGCGTTTCAGAGCTATGCTGGAAA
TkR99D	2	CCCGTCGCCACAATGACCATTGCACCAGCCGGGAATCGAACC
TkR99D	3	ATGGTCATTGTGGCGACGGGGTTTCAGAGCTATGCTGGAAAC
TkR99D	4	TTTAACTTGCTATTTCTAGCTCTAAAACAAGGGCCAGTCGCTATCCAGTGCACCAGCCGGGAATCGAAC
TkR86C	1	GGCCCGGGTTCGATTCCCGGCCGATGCAGTCGAGCATTATCGACAATCGTTTCAGAGCTATGCTGGAAA
TkR86C	2	ATCTGAGTTCAGCATGAATATGCACCAGCCGGGAATCGAACC

TkR86C	3	TATTCATGCTGAACTCAGATGTTTCAGAGCTATGCTGGAAAC
TkR86C	4	TTTAACTTGCTATTTCTAGCTCTAAAACATTGATCTACTGCCCCACAGTGCACCAGCCGGGAATCGAAC
Lkr	1	GGCCCGGGTTCGATTCCCGGCCGATGCAGGAATTCCTGCCCGGAGCCGGTTTCAGAGCTATGCTGGAAA
Lkr	2	GGTCGTGGCCACCACCCAGATGCACCAGCCGGGAATCGAACC
Lkr	3	TCTGGGTGGTGGCCACGACCGTTTCAGAGCTATGCTGGAAAC
Lkr	4	TTTAACTTGCTATTTCTAGCTCTAAAACGGACGAAGGGGCAGAAGCTGTGCACCAGCCGGGAATCGAAC
Rya-R	1	GGCCCGGGTTCGATTCCCGGCCGATGCAGCAGCCGATGCTGCGGAACGGTTTCAGAGCTATGCTGGAAA
Rya-R	2	GCGAGTAGTTCACAAAGTGATGCACCAGCCGGGAATCGAACC
Rya-R	3	TCACTTTGTGAACTACTCGCGTTTCAGAGCTATGCTGGAAAC
Rya-R	4	TTTAACTTGCTATTTCTAGCTCTAAAACAGCGGCACGACGAACTGCAGTGCACCAGCCGGGAATCGAAC
SIFaR	1	GGCCCGGGTTCGATTCCCGGCCGATGCACCATTCCGACCATGGCGCCGGTTTCAGAGCTATGCTGGAAA
SIFaR	2	AGGCTGCCACGGAGACACCTTGCACCAGCCGGGAATCGAACC
SIFaR	3	AGGTGTCTCCGTGGCAGCCTGTTTCAGAGCTATGCTGGAAAC
SIFaR	4	TTTAACTTGCTATTTCTAGCTCTAAAACGGGCGTCGGAGAAGACCTCCTGCACCAGCCGGGAATCGAAC
CCKLR-17D1	1	GGCCCGGGTTCGATTCCCGGCCGATGCACGCGGAACCCGAACTCCATAGTTTCAGAGCTATGCTGGAAA
CCKLR-17D1	2	GGTGCGCGACCTCAGCGGGTTGCACCAGCCGGGAATCGAACC
CCKLR-17D1	3	ACCCGCTGAGGTCGCGCACCGTTTCAGAGCTATGCTGGAAAC
CCKLR-17D1	4	TTTAACTTGCTATTTCTAGCTCTAAAACCCGGCCCACTGCTGCCAAAGTGCACCAGCCGGGAATCGAAC
CCKLR-17D3	1	GGCCCGGGTTCGATTCCCGGCCGATGCATATGGCGATGATGATAGGGAGTTTCAGAGCTATGCTGGAAA
CCKLR-17D3	2	AGGTCCAGGACGAAACGGCCTGCACCAGCCGGGAATCGAACC
CCKLR-17D3	3	GGCCGTTTCGTCCTGGACCTGTTTCAGAGCTATGCTGGAAAC
CCKLR-17D3	4	TTTAACTTGCTATTTCTAGCTCTAAAACCGCAGAGGACGAGAAGCGGCTGCACCAGCCGGGAATCGAAC
sNPF-R	1	GGCCCGGGTTCGATTCCCGGCCGATGCAGGGAACTACTAGCGCTATCTGTTTCAGAGCTATGCTGGAAA
sNPF-R	2	TGAACGTGTAAAGCGGAGTATGCACCAGCCGGGAATCGAACC
sNPF-R	3	TACTCCGCTTTACACGTTCAGTTTCAGAGCTATGCTGGAAAC
sNPF-R	4	TTTAACTTGCTATTTCTAGCTCTAAAACCCTTGCCATGATGCAGTCGCTGCACCAGCCGGGAATCGAAC
NPFR	1	GGCCCGGGTTCGATTCCCGGCCGATGCAGCAGATGGGGAGCATCTGAGGTTTCAGAGCTATGCTGGAAA
NPFR	2	GCGGCCTGCTCCCAGTCTCATGCACCAGCCGGGAATCGAACC
NPFR	3	TGAGACTGGGAGCAGGCCGCGTTTCAGAGCTATGCTGGAAAC
NPFR	4	TTTAACTTGCTATTTCTAGCTCTAAAACTAGGGCCAGTACTTGGACAGTGCACCAGCCGGGAATCGAAC
Dh31-R	1	GGCCCGGGTTCGATTCCCGGCCGATGCATTCCGCCTACGAACTCTGCCGTTTCAGAGCTATGCTGGAAA
Dh31-R	2	GCCTCTTCTCGGAAATGAATTGCACCAGCCGGGAATCGAACC

Dh31-R	3	ATTCATTTCCGAGAAGAGGCGTTTCAGAGCTATGCTGGAAAC
Dh31-R	4	TTTAACTTGCTATTTCTAGCTCTAAAACTGAATACTGGCCGGGGCATTTGCACCAGCCGGGAATCGAAC
hec	1	GGCCCGGGTTCGATTCCCGGCCGATGCAAAATGTGGATGTGGCATCGCGTTTCAGAGCTATGCTGGAAA
hec	2	CCAGGTCCTCATAGTCCACGTGCACCAGCCGGGAATCGAACC
hec	3	CGTGGACTATGAGGACCTGGGTTTCAGAGCTATGCTGGAAAC
hec	4	TTTAACTTGCTATTTCTAGCTCTAAAACGCAGGGAGAGGGCATAACCCTGCACCAGCCGGGAATCGAAC
Dh44-R2	1	GGCCCGGGTTCGATTCCCGGCCGATGCAAGACGACGATTTGAGGGCACGTTTCAGAGCTATGCTGGAAA
Dh44-R2	2	GACTGCCGGCGTTTGTGCGTTGCACCAGCCGGGAATCGAACC
Dh44-R2	3	ACGCACAAACGCCGGCAGTCGTTTCAGAGCTATGCTGGAAAC
Dh44-R2	4	TTTAACTTGCTATTTCTAGCTCTAAAACACGTTCCGTTTGGAAAGCAATGCACCAGCCGGGAATCGAAC
Dh44-R1	1	GGCCCGGGTTCGATTCCCGGCCGATGCATCGATTCGGTGAACGCCAGCGTTTCAGAGCTATGCTGGAAA
Dh44-R1	2	TTGGCGTGGCAGAACCTCGTTGCACCAGCCGGGAATCGAACC
Dh44-R1	3	ACGAGGTTCTGCCACGCCAAGTTTCAGAGCTATGCTGGAAAC
Dh44-R1	4	TTTAACTTGCTATTTCTAGCTCTAAAACCTGTATTGGCCGAGCGCAGCTGCACCAGCCGGGAATCGAAC
PDFR	1	GGCCCGGGTTCGATTCCCGGCCGATGCATCGAACATTCTCGACTGCGGGTTTCAGAGCTATGCTGGAAA
PDFR	2	GCCGGAGTGGGTGGCCAGCATGCACCAGCCGGGAATCGAACC
PDFR	3	TGCTGGCCACCCACTCCGGCGTTTCAGAGCTATGCTGGAAAC
PDFR	4	TTTAACTTGCTATTTCTAGCTCTAAAACCCTGGCAATGTCTATGTAGGTGCACCAGCCGGGAATCGAAC
CG11318	1	GGCCCGGGTTCGATTCCCGGCCGATGCATGGAGAGGAACGTAATTCATGTTTCAGAGCTATGCTGGAAA
CG11318	2	TGTCCTGCTGCATCAGGGCGTGCACCAGCCGGGAATCGAACC
CG11318	3	CGCCCTGATGCAGCAGGACAGTTTCAGAGCTATGCTGGAAAC
CG11318	4	TTTAACTTGCTATTTCTAGCTCTAAAACTCAGTTGACCAGGTTTCATATGCACCAGCCGGGAATCGAAC
CG15556	1	GGCCCGGGTTCGATTCCCGGCCGATGCAGCTGTCGCACAATCCCTACAGTTTCAGAGCTATGCTGGAAA
CG15556	2	GAGTTTGAGTCAGGAGGTTATGCACCAGCCGGGAATCGAACC
CG15556	3	TAACCTCCTGACTCAAACTCGTTTCAGAGCTATGCTGGAAAC
CG15556	4	TTTAACTTGCTATTTCTAGCTCTAAAACTCGTTTGATTTCCTCTTCCGTGCACCAGCCGGGAATCGAAC
5-HT1B	1	GGCCCGGGTTCGATTCCCGGCCGATGCAATCGCCATGGCCGTGGTGCTGTTTCAGAGCTATGCTGGAAA
5-HT1B	2	GGCAATAATGTAGATTTTCCTGCACCAGCCGGGAATCGAACC
5-HT1B	3	GGAAAATCTACATTATTGCCGTTTCAGAGCTATGCTGGAAAC
5-HT1B	4	TTTAACTTGCTATTTCTAGCTCTAAAACCCCGTCCCGTCGCCGTCCACTGCACCAGCCGGGAATCGAAC
5-HT1A	1	GGCCCGGGTTCGATTCCCGGCCGATGCATAGCGAACAGCATGAATGACGTTTCAGAGCTATGCTGGAAA
5-HT1A	2	GGATAATGGCCGCTATGACATGCACCAGCCGGGAATCGAACC

5-HT1A	3	TGTCATAGCGGCCATTATCCGTTTCAGAGCTATGCTGGAAAC
5-HT1A	4	TTTAACTTGCTATTTCTAGCTCTAAAACCATCGACGGGTCGCGGTCGTTGCACCAGCCGGGAATCGAAC
5-HT7	1	GGCCCGGGTTCGATTCCCGGCCGATGCACACAGAAACCACAGAACCCAGTTTCAGAGCTATGCTGGAAA
5-HT7	2	AAATTGCTGCTGGTGATGCTGCACCAGCCGGGAATCGAACC
5-HT7	3	GCATCACCAGCAGCAATTTGTTTCAGAGCTATGCTGGAAAC
5-HT7	4	TTTAACTTGCTATTTCTAGCTCTAAAACGAAGAGCCACACAGAGATCCTGCACCAGCCGGGAATCGAAC
mAChR-A	1	GGCCCGGGTTCGATTCCCGGCCGATGCAAAAGGGACCTCGGTACTCACGTTTCAGAGCTATGCTGGAAA
mAChR-A	2	CGTCCGGTGAACTCTCGGAGTGCACCAGCCGGGAATCGAACC
mAChR-A	3	CTCCGAGAGTTCACCGGACGGTTTCAGAGCTATGCTGGAAAC
mAChR-A	4	TTTAACTTGCTATTTCTAGCTCTAAAACGCTCGTAGGCGAAGTCGTCGTGCACCAGCCGGGAATCGAAC
mAChR-B	1	GGCCCGGGTTCGATTCCCGGCCGATGCAGCGGTCGCTTAACAAGTCGGGTTTCAGAGCTATGCTGGAAA
mAChR-B	2	TGTGCGTCAGGACTCGCTTGTGCACCAGCCGGGAATCGAACC
mAChR-B	3	CAAGCGAGTCCTGACGCACAGTTTCAGAGCTATGCTGGAAAC
mAChR-B	4	TTTAACTTGCTATTTCTAGCTCTAAAACTGAGCACGTATATCGTGTAGTGCACCAGCCGGGAATCGAAC
Oct-TyrR	1	GGCCCGGGTTCGATTCCCGGCCGATGCAGTTTGTAAATGTCACCACAAGTTTCAGAGCTATGCTGGAAA
Oct-TyrR	2	AGAGAACCAGGGCGGTGAGATGCACCAGCCGGGAATCGAACC
Oct-TyrR	3	TCTCACCGCCCTGGTTCTCTGTTTCAGAGCTATGCTGGAAAC
Oct-TyrR	4	TTTAACTTGCTATTTCTAGCTCTAAAACGCACGACCACGTGCTGCTGTTGCACCAGCCGGGAATCGAAC
Oamb	1	GGCCCGGGTTCGATTCCCGGCCGATGCAACCAGCCAATCTGATCTCCCGTTTCAGAGCTATGCTGGAAA
Oamb	2	CCACATAGCGGTCCAGTGATTGCACCAGCCGGGAATCGAACC
Oamb	3	ATCACTGGACCGCTATGTGGGTTTCAGAGCTATGCTGGAAAC
Oamb	4	TTTAACTTGCTATTTCTAGCTCTAAAACCGCCCTGGTCCTTAATGCTATGCACCAGCCGGGAATCGAAC
Dop1R2	1	GGCCCGGGTTCGATTCCCGGCCGATGCACCTCCTGGCGCTCCTCCGGGGTTTCAGAGCTATGCTGGAAA
Dop1R2	2	GATAGCCTAGGTGCTCAGTGTGCACCAGCCGGGAATCGAACC
Dop1R2	3	CACTGAGCACCTAGGCTATCGTTTCAGAGCTATGCTGGAAAC
Dop1R2	4	TTTAACTTGCTATTTCTAGCTCTAAAACGCCGAGCCAGGTGACGATTGTGCACCAGCCGGGAATCGAAC
CG18208	1	GGCCCGGGTTCGATTCCCGGCCGATGCACATCAGCACCGATGACTTTCGTTTCAGAGCTATGCTGGAAA
CG18208	2	GTAGCCCATTAGCTCATTGGTGCACCAGCCGGGAATCGAACC
CG18208	3	CCAATGAGCTAATGGGCTACGTTTCAGAGCTATGCTGGAAAC
CG18208	4	TTTAACTTGCTATTTCTAGCTCTAAAACCGCCCGCGCCTTGGCGGCAATGCACCAGCCGGGAATCGAAC
TyrRII	1	GGCCCGGGTTCGATTCCCGGCCGATGCATGTTGTCAACAGCCCTCACTGGTTTCAGAGCTATGCTGGAAA
TyrRII	2	CTGCAGAGCAGGATGTCCAGTGCACCAGCCGGGAATCGAACC

TyrRII	3	CTGGACATCCTGCTCTGCAGGTTTCAGAGCTATGCTGGAAAC
TyrRII	4	TTTAACTTGCTATTTCTAGCTCTAAAACCACGACGAGAATCATGAGGATGCACCAGCCGGGAATCGAAC
TyrR	1	GGCCCGGGTTCGATTCCCGGCCGATGCATTATCCGTCGCTCATGTATGGTTTCAGAGCTATGCTGGAAA
TyrR	2	CGAGAAGGTCGGCCACCGCCTGCACCAGCCGGGAATCGAACC
TyrR	3	GGCGGTGGCCGACCTTCTCGGTTTCAGAGCTATGCTGGAAAC
TyrR	4	TTTAACTTGCTATTTCTAGCTCTAAAACATCGTTTGGAGCGCCGTTTATGCACCAGCCGGGAATCGAAC
Dop2R	1	GGCCCGGGTTCGATTCCCGGCCGATGCAGGCGGCAACGGGAGCAACTGGTTTCAGAGCTATGCTGGAAA
Dop2R	2	AAACCGTGCTCCCATTGTAGTGCACCAGCCGGGAATCGAACC
Dop2R	3	CTACAATGGGAGCACGGTTTGTTTCAGAGCTATGCTGGAAAC
Dop2R	4	TTTAACTTGCTATTTCTAGCTCTAAAACAAGAGGGTCAGGATGGGGAATGCACCAGCCGGGAATCGAAC
Dop1R1	1	GGCCCGGGTTCGATTCCCGGCCGATGCAAATCACACCAGTGGGAGCACGTTTCAGAGCTATGCTGGAAA
Dop1R1	2	ACTATTGTATCCATTTCAGATGCACCAGCCGGGAATCGAACC
Dop1R1	3	TCTGAAATGGATACAATAGTGTTTCAGAGCTATGCTGGAAAC
Dop1R1	4	TTTAACTTGCTATTTCTAGCTCTAAAACGCGCAGGCTGCGCTCCGTGTTGCACCAGCCGGGAATCGAAC
Octbeta2R	1	GGCCCGGGTTCGATTCCCGGCCGATGCAGCCCGGAGCCACCGCGGCAAGTTTCAGAGCTATGCTGGAAA
Octbeta2R	2	GTTACTTGCACACTAAAGTTTGCACCAGCCGGGAATCGAACC
Octbeta2R	3	AACTTTAGTGTGCAAGTAACGTTTCAGAGCTATGCTGGAAAC
Octbeta2R	4	TTTAACTTGCTATTTCTAGCTCTAAAACCAAATCGCACAGGAAGGGGCTGCACCAGCCGGGAATCGAAC
Octbeta1R	1	GGCCCGGGTTCGATTCCCGGCCGATGCACACGACCAGGACAATACTGGGTTTCAGAGCTATGCTGGAAA
Octbeta1R	2	AGATCATGACGGAAGCATTATGCACCAGCCGGGAATCGAACC
Octbeta1R	3	TAATGCTTCCGTCATGATCTGTTTCAGAGCTATGCTGGAAAC
Octbeta1R	4	TTTAACTTGCTATTTCTAGCTCTAAAACCACCATCAATAGCATGATGATGCACCAGCCGGGAATCGAAC
Octbeta3R	1	GGCCCGGGTTCGATTCCCGGCCGATGCAGCCTATAACATCGCAATTTGGTTTCAGAGCTATGCTGGAAA
Octbeta3R	2	GGCTGTTGTACACGTTGCACTGCACCAGCCGGGAATCGAACC
Octbeta3R	3	GTGCAACGTGTACAACAGCCGTTTCAGAGCTATGCTGGAAAC
Octbeta3R	4	TTTAACTTGCTATTTCTAGCTCTAAAACGATGCAGGGAAATCTCCCGCTGCACCAGCCGGGAATCGAAC
5-HT2B	1	GGCCCGGGTTCGATTCCCGGCCGATGCAGAGGATGTGTATGCCTCGCTGTTTCAGAGCTATGCTGGAAA
5-HT2B	2	CGGAGATGGTGCACAGGTGCTGCACCAGCCGGGAATCGAACC
5-HT2B	3	GCACCTGTGCACCATCTCCGGTTTCAGAGCTATGCTGGAAAC
5-HT2B	4	TTTAACTTGCTATTTCTAGCTCTAAAACGTATCTGGCAAGTTCCATTCTGCACCAGCCGGGAATCGAAC
5-HT2A	1	GGCCCGGGTTCGATTCCCGGCCGATGCACAGCCGTTTATTGACTGCGAGTTTCAGAGCTATGCTGGAAA
5-HT2A	2	TGGGGACTGTAGTCCAGAATGCACCAGCCGGGAATCGAACC

5-HT2A	3	TTCTGGACTACAGTCCCCAGTTTCAGAGCTATGCTGGAAAC
5-HT2A	4	TTTAACTTGCTATTTCTAGCTCTAAAACAACTGATGAAGCACATGTGCTGCACCAGCCGGGAATCGAAC
AdoR	1	GGCCCGGGTTCGATTCCCGGCCGATGCAGATCACCGATTTCTCCTTCGGTTTCAGAGCTATGCTGGAAA
AdoR	2	GGGATACTATGTAGTAGTTGTGCACCAGCCGGGAATCGAACC
AdoR	3	CAACTACTACATAGTATCCCGTTTCAGAGCTATGCTGGAAAC
AdoR	4	TTTAACTTGCTATTTCTAGCTCTAAAACTGCGGGTGCGGACATTCCTTTGCACCAGCCGGGAATCGAAC
mAchR-C	1	GGCCCGGGTTCGATTCCCGGCCGATGCATGGGTGTCGTCCCGTTCCTGGTTTCAGAGCTATGCTGGAAA
mAchR-C	2	TCTATAGTGCAGAGCATAGATGCACCAGCCGGGAATCGAACC
mAchR-C	3	TCTATGCTCTGCACTATAGAGTTTCAGAGCTATGCTGGAAAC
mAchR-C	4	TTTAACTTGCTATTTCTAGCTCTAAAACGTCTGGATGGAGCAGCAGATTGCACCAGCCGGGAATCGAAC
CG13579	1	GGCCCGGGTTCGATTCCCGGCCGATGCATGCTCGCATCCGCGCTACTCGTTTCAGAGCTATGCTGGAAA
CG13579	2	TGCAAACCAGGATGACGGCGTGCACCAGCCGGGAATCGAACC
CG13579	3	CGCCGTCATCCTGGTTTGCAGTTTCAGAGCTATGCTGGAAAC
CG13579	4	TTTAACTTGCTATTTCTAGCTCTAAAACGAAGCGGCTCATGTGGAAGCTGCACCAGCCGGGAATCGAAC
DopEcR	1	GGCCCGGGTTCGATTCCCGGCCGATGCAGCTCATATCGATACTGGGCGGTTTCAGAGCTATGCTGGAAA
DopEcR	2	GTCAAAGCGGGATACACGGATGCACCAGCCGGGAATCGAACC
DopEcR	3	TCCGTGTATCCCGCTTTGACGTTTCAGAGCTATGCTGGAAAC
DopEcR	4	TTTAACTTGCTATTTCTAGCTCTAAAACTATTCCCCCAGTCCAGCATGTGCACCAGCCGGGAATCGAAC
Cirl	1	GGCCCGGGTTCGATTCCCGGCCGATGCACAAGCAGAGCTGCGGCGTGTGTTTCAGAGCTATGCTGGAAA
Cirl	2	AATCTGGGAAGACCACGCTGTGCACCAGCCGGGAATCGAACC
Cirl	3	CAGCGTGGTCTTCCCAGATTGTTTCAGAGCTATGCTGGAAAC
Cirl	4	TTTAACTTGCTATTTCTAGCTCTAAAACCGCGTTGACATCGCTGTCGTTGCACCAGCCGGGAATCGAAC
stan	1	GGCCCGGGTTCGATTCCCGGCCGATGCAAAACGAGGGAGTTCCCTCAAGTTTCAGAGCTATGCTGGAAA
stan	2	TGCTCTGGTGCTCGATGGCATGCACCAGCCGGGAATCGAACC
stan	3	TGCCATCGAGCACCAGAGCAGTTTCAGAGCTATGCTGGAAAC
stan	4	TTTAACTTGCTATTTCTAGCTCTAAAACACGGGAACATTCTCTAGCACTGCACCAGCCGGGAATCGAAC
mthl14	1	GGCCCGGGTTCGATTCCCGGCCGATGCAACTGTAAATAACAGTTCGAAGTTTCAGAGCTATGCTGGAAA
mthl14	2	TCACAGTGTATGATTCGTTGTGCACCAGCCGGGAATCGAACC
mthl14	3	CAACGAATCATACACTGTGAGTTTCAGAGCTATGCTGGAAAC
mthl14	4	TTTAACTTGCTATTTCTAGCTCTAAAACAGCACAGAGACATCAAACTTTGCACCAGCCGGGAATCGAAC
mthl5	1	GGCCCGGGTTCGATTCCCGGCCGATGCAGTCATCGGGATTCCAAATTGGTTTCAGAGCTATGCTGGAAA
mthl5	2	CATAATCCGATTGTATTCCATGCACCAGCCGGGAATCGAACC

mthl5	3	TGGAATACAATCGGATTATGGTTTCAGAGCTATGCTGGAAAC
mthl5	4	TTTAACTTGCTATTTCTAGCTCTAAAACGCCGTCCGTGACTCGCAGGATGCACCAGCCGGGAATCGAAC
mthl9	1	GGCCCGGGTTCGATTCCCGGCCGATGCATCCCTGTGCCTACGCCCACAGTTTCAGAGCTATGCTGGAAA
mthl9	2	GCAACCCAGCTCTGTGCGCATGCACCAGCCGGGAATCGAACC
mthl9	3	TGCGCACAGAGCTGGGTTGCGTTTCAGAGCTATGCTGGAAAC
mthl9	4	TTTAACTTGCTATTTCTAGCTCTAAAACACGATCATGGACAGCAAGTATGCACCAGCCGGGAATCGAAC
mthl8	1	GGCCCGGGTTCGATTCCCGGCCGATGCATTCTGGAACACACTGCTCGTGTTTCAGAGCTATGCTGGAAA
mthl8	2	ATGGAGCAGCGGAGTGAAGCTGCACCAGCCGGGAATCGAACC
mthl8	3	GCTTCACTCCGCTGCTCCATGTTTCAGAGCTATGCTGGAAAC
mthl8	4	TTTAACTTGCTATTTCTAGCTCTAAAACTCAGCACGATAAACATTGATTGCACCAGCCGGGAATCGAAC
mthl1	1	GGCCCGGGTTCGATTCCCGGCCGATGCAGTCGATGAGTTTGGCCATCGGTTTCAGAGCTATGCTGGAAA
mthl1	2	CACATAGAGCGTGCCATTGGTGCACCAGCCGGGAATCGAACC
mthl1	3	CCAATGGCACGCTCTATGTGGTTTCAGAGCTATGCTGGAAAC
mthl1	4	TTTAACTTGCTATTTCTAGCTCTAAAACAAGTGGCGGACAGGAAGACATGCACCAGCCGGGAATCGAAC
mthl15	1	GGCCCGGGTTCGATTCCCGGCCGATGCATCGCCGCACTGTGGACAATGGTTTCAGAGCTATGCTGGAAA
mthl15	2	GGTTTGCATGTATTATTTTCTGCACCAGCCGGGAATCGAACC
mthl15	3	GAAAATAATACATGCAAACCGTTTCAGAGCTATGCTGGAAAC
mthl15	4	TTTAACTTGCTATTTCTAGCTCTAAAACTCGGCACAACAACGACGAATTGCACCAGCCGGGAATCGAAC
mthl13	1	GGCCCGGGTTCGATTCCCGGCCGATGCACGGAAAAATATGATGGCCAAGTTTCAGAGCTATGCTGGAAA
mthl13	2	TAAGCCAGATTAGATAGTCATGCACCAGCCGGGAATCGAACC
mthl13	3	TGACTATCTAATCTGGCTTAGTTTCAGAGCTATGCTGGAAAC
mthl13	4	TTTAACTTGCTATTTCTAGCTCTAAAACTATGCAATAGCTGATTATGGTGCACCAGCCGGGAATCGAAC
mthl10	1	GGCCCGGGTTCGATTCCCGGCCGATGCATCCCTTCGGCACTTACGTTAGTTTCAGAGCTATGCTGGAAA
mthl10	2	ATATCGTAGACTCGTTGCGGTGCACCAGCCGGGAATCGAACC
mthl10	3	CCGCAACGAGTCTACGATATGTTTCAGAGCTATGCTGGAAAC
mthl10	4	TTTAACTTGCTATTTCTAGCTCTAAAACACGGAGAGCCCTATTAAATATGCACCAGCCGGGAATCGAAC
mthl11	1	GGCCCGGGTTCGATTCCCGGCCGATGCACTCTTAGGTATCCTTGTGATGTTTCAGAGCTATGCTGGAAA
mthl11	2	GGGTGTATTCGTAGGTAAGGTGCACCAGCCGGGAATCGAACC
mthl11	3	CCTTACCTACGAATACACCCGTTTCAGAGCTATGCTGGAAAC
mthl11	4	TTTAACTTGCTATTTCTAGCTCTAAAACTTACAGTGAAGTAGCCAGCATGCACCAGCCGGGAATCGAAC
mthl6	1	GGCCCGGGTTCGATTCCCGGCCGATGCACAATCGGAGGCAGTTATTCCGTTTCAGAGCTATGCTGGAAA
mthl6	2	GTGTGATCTTCAAGTAGGGATGCACCAGCCGGGAATCGAACC

mthl6	3	TCCCTACTTGAAGATCACACGTTTCAGAGCTATGCTGGAAAC
mthl6	4	TTTAACTTGCTATTTCTAGCTCTAAAACTATTTGATGACTCATAACAGTGCACCAGCCGGGAATCGAAC
mthl7	1	GGCCCGGGTTCGATTCCCGGCCGATGCAGTCTCAAAGAGGAGCTCGCCGTTTCAGAGCTATGCTGGAAA
mthl7	2	CGAAACTATACAGCATACAATGCACCAGCCGGGAATCGAACC
mthl7	3	TTGTATGCTGTATAGTTTCGGTTTCAGAGCTATGCTGGAAAC
mthl7	4	TTTAACTTGCTATTTCTAGCTCTAAAACATAGTTAGGGTCGACTCGATTGCACCAGCCGGGAATCGAAC
mthl12	1	GGCCCGGGTTCGATTCCCGGCCGATGCAGAAATTCCTGCCAACCTGACGTTTCAGAGCTATGCTGGAAA
mthl12	2	ACATGGTTAGTTTAATTTCATGCACCAGCCGGGAATCGAACC
mthl12	3	TGAAATTAAACTAACCATGTGTTTCAGAGCTATGCTGGAAAC
mthl12	4	TTTAACTTGCTATTTCTAGCTCTAAAACAGTAGATTTCGGGATAAAGATGCACCAGCCGGGAATCGAAC
mth	1	GGCCCGGGTTCGATTCCCGGCCGATGCATTTCGACACTGTCGATATTTGTTTCAGAGCTATGCTGGAAA
mth	2	TGATATGGTCGTGAGGGCAGTGCACCAGCCGGGAATCGAACC
mth	3	CTGCCCTCACGACCATATCAGTTTCAGAGCTATGCTGGAAAC
mth	4	TTTAACTTGCTATTTCTAGCTCTAAAACCTTGCGAAGAGTCACACGGTTGCACCAGCCGGGAATCGAAC
mthl2	1	GGCCCGGGTTCGATTCCCGGCCGATGCAGATACCGTTGATATCTCGGAGTTTCAGAGCTATGCTGGAAA
mthl2	2	TCGTCATGTTGGCGTAGCATTGCACCAGCCGGGAATCGAACC
mthl2	3	ATGCTACGCCAACATGACGAGTTTCAGAGCTATGCTGGAAAC
mthl2	4	TTTAACTTGCTATTTCTAGCTCTAAAACATAGTGCCGACTGATGATAGTGCACCAGCCGGGAATCGAAC
mthl3	1	GGCCCGGGTTCGATTCCCGGCCGATGCATCTTACAATTTATACGCCTGGTTTCAGAGCTATGCTGGAAA
mthl3	2	GAGGGTAAGACTGATGACAGTGCACCAGCCGGGAATCGAACC
mthl3	3	CTGTCATCAGTCTTACCCTCGTTTCAGAGCTATGCTGGAAAC
mthl3	4	TTTAACTTGCTATTTCTAGCTCTAAAACAATTGAGCCTATTGGTTGTTTGCACCAGCCGGGAATCGAAC
mthl4	1	GGCCCGGGTTCGATTCCCGGCCGATGCACTACTTGGCCTCTTTGTTCTGTTTCAGAGCTATGCTGGAAA
mthl4	2	CAGGCGTATAAGTTGTATGCTGCACCAGCCGGGAATCGAACC
mthl4	3	GCATACAACTTATACGCCTGGTTTCAGAGCTATGCTGGAAAC
mthl4	4	TTTAACTTGCTATTTCTAGCTCTAAAACAAAGGACAAACATTATTATGTGCACCAGCCGGGAATCGAAC
ninaE	1	GGCCCGGGTTCGATTCCCGGCCGATGCACTGGAACCAGTTCCCCGCCAGTTTCAGAGCTATGCTGGAAA
ninaE	2	AGCCCAGTCCGGCGTATATGTGCACCAGCCGGGAATCGAACC
ninaE	3	CATATACGCCGGACTGGGCTGTTTCAGAGCTATGCTGGAAAC
ninaE	4	TTTAACTTGCTATTTCTAGCTCTAAAACCACGAGGTCAGGTTACCCTCTGCACCAGCCGGGAATCGAAC
rh2	1	GGCCCGGGTTCGATTCCCGGCCGATGCAGCCGGAGACGCCATTCGACCGTTTCAGAGCTATGCTGGAAA
rh2	2	AGTCTCGTAGTAGAAGTTAATGCACCAGCCGGGAATCGAACC

rh2	3	TTAACTTCTACTACGAGACTGTTTCAGAGCTATGCTGGAAAC
rh2	4	TTTAACTTGCTATTTCTAGCTCTAAAACCAGGGATTTCACATTCATCTTGCACCAGCCGGGAATCGAAC
rh3	1	GGCCCGGGTTCGATTCCCGGCCGATGCAGCCGGAGACGCCATTCGACCGTTTCAGAGCTATGCTGGAAA
rh3	2	TGGTGGAAGCTATTGTAGATTGCACCAGCCGGGAATCGAACC
rh3	3	ATCTACAATAGCTTCCACCAGTTTCAGAGCTATGCTGGAAAC
rh3	4	TTTAACTTGCTATTTCTAGCTCTAAAACCAAAGAGTCGCGTATCGAAGTGCACCAGCCGGGAATCGAAC
rh4	1	GGCCCGGGTTCGATTCCCGGCCGATGCATTCAATACATTCCGGAGCACGTTTCAGAGCTATGCTGGAAA
rh4	2	AGGGCGAATCCTCGATGGAATGCACCAGCCGGGAATCGAACC
rh4	3	TTCCATCGAGGATTCGCCCTGTTTCAGAGCTATGCTGGAAAC
rh4	4	TTTAACTTGCTATTTCTAGCTCTAAAACCGATCTGCGAGTAGTAGTAATGCACCAGCCGGGAATCGAAC
rh5	1	GGCCCGGGTTCGATTCCCGGCCGATGCATGCACATCAACGGCCCAAGTGTTTCAGAGCTATGCTGGAAA
rh5	2	CCGTTCAAAGCGTATATATCTGCACCAGCCGGGAATCGAACC
rh5	3	GATATATACGCTTTGAACGGGTTTCAGAGCTATGCTGGAAAC
rh5	4	TTTAACTTGCTATTTCTAGCTCTAAAACTGTTGGTCAGGTAATCGAAGTGCACCAGCCGGGAATCGAAC
rh6	1	GGCCCGGGTTCGATTCCCGGCCGATGCACCATTGGAGCCCATGTGGTTGTTTCAGAGCTATGCTGGAAA
rh6	2	TATAATTCGCACAGGAAGGGTGCACCAGCCGGGAATCGAACC
rh6	3	CCCTTCCTGTGCGAATTATAGTTTCAGAGCTATGCTGGAAAC
rh6	4	TTTAACTTGCTATTTCTAGCTCTAAAACCGTAGTTGATGATGGTATACTGCACCAGCCGGGAATCGAAC
rh7	1	GGCCCGGGTTCGATTCCCGGCCGATGCAGAATCATCCGCTGTGAATGTGTTTCAGAGCTATGCTGGAAA
rh7	2	TGTAGTGAATGTAGCTGGGGTGCACCAGCCGGGAATCGAACC
rh7	3	CCCCAGCTACATTCACTACAGTTTCAGAGCTATGCTGGAAAC
rh7	4	TTTAACTTGCTATTTCTAGCTCTAAAACATCCAAAGCGATGGCGGTGATGCACCAGCCGGGAATCGAAC
mGluR	1	GGCCCGGGTTCGATTCCCGGCCGATGCATCAGTGTCTGTTTCTCTGCCGTTTCAGAGCTATGCTGGAAA
mGluR	2	CGAGCGTTAGGCTTCTTTTGTGCACCAGCCGGGAATCGAACC
mGluR	3	CAAAAGAAGCCTAACGCTCGGTTTCAGAGCTATGCTGGAAAC
mGluR	4	TTTAACTTGCTATTTCTAGCTCTAAAACTGTCTCAGTCGTTTGGTCGCTGCACCAGCCGGGAATCGAAC
mtt	1	GGCCCGGGTTCGATTCCCGGCCGATGCACATCAAGCTCACGTTGGTAAGTTTCAGAGCTATGCTGGAAA
mtt	2	CCCCCGAGATGACCTTGCGCTGCACCAGCCGGGAATCGAACC
mtt	3	GCGCAAGGTCATCTCGGGGGGTTTCAGAGCTATGCTGGAAAC
mtt	4	TTTAACTTGCTATTTCTAGCTCTAAAACCGGCTTCGTCAGCAGCTTCTTGCACCAGCCGGGAATCGAAC
GABA-B-R1	1	GGCCCGGGTTCGATTCCCGGCCGATGCAATCGCCTCGCCGCACCTGCAGTTTCAGAGCTATGCTGGAAA
GABA-B-R1	2	GGCGTCGCAAATTGCGCACGTGCACCAGCCGGGAATCGAACC

GABA-B-R1	3	CGTGCGCAATTTGCGACGCCGTTTCAGAGCTATGCTGGAAAC
GABA-B-R1	4	TTTAACTTGCTATTTCTAGCTCTAAAACGTGGATAGTAACCAAGCCCGTGCACCAGCCGGGAATCGAAC
GABA-B-R2	1	GGCCCGGGTTCGATTCCCGGCCGATGCAGAAAACTCCTACACCGGTCGGTTTCAGAGCTATGCTGGAAA
GABA-B-R2	2	GGGCAGCTGCCCAGATACCATGCACCAGCCGGGAATCGAACC
GABA-B-R2	3	TGGTATCTGGGCAGCTGCCCGTTTCAGAGCTATGCTGGAAAC
GABA-B-R2	4	TTTAACTTGCTATTTCTAGCTCTAAAACTTCGATCCGATAGCACCAGGTGCACCAGCCGGGAATCGAAC
GABA-B-R3	1	GGCCCGGGTTCGATTCCCGGCCGATGCACCGGTCCAAGGGACCAGATAGTTTCAGAGCTATGCTGGAAA
GABA-B-R3	2	CGTCGAGGGCTGTGTGTAGATGCACCAGCCGGGAATCGAACC
GABA-B-R3	3	TCTACACACAGCCCTCGACGGTTTCAGAGCTATGCTGGAAAC
GABA-B-R3	4	TTTAACTTGCTATTTCTAGCTCTAAAACGTCGCTCAACGCCGGCGATGTGCACCAGCCGGGAATCGAAC
fz	1	GGCCCGGGTTCGATTCCCGGCCGATGCATTTACCCACCCTGATACAGGGTTTCAGAGCTATGCTGGAAA
fz	2	TTCTCTCTCCGGGAAGAACATGCACCAGCCGGGAATCGAACC
fz	3	TGTTCTTCCCGGAGAGAGAAGTTTCAGAGCTATGCTGGAAAC
fz	4	TTTAACTTGCTATTTCTAGCTCTAAAACCGTAGGCACATCCAACTACCTGCACCAGCCGGGAATCGAAC
fz2	1	GGCCCGGGTTCGATTCCCGGCCGATGCACGGAGTGCCAGTCATACCCAGTTTCAGAGCTATGCTGGAAA
fz2	2	TCCCCAGGAAGATGAGTGGGTGCACCAGCCGGGAATCGAACC
fz2	3	CCCACTCATCTTCCTGGGGAGTTTCAGAGCTATGCTGGAAAC
fz2	4	TTTAACTTGCTATTTCTAGCTCTAAAACTGTAGAGCACCGAGAAGATGTGCACCAGCCGGGAATCGAAC
fz3	1	GGCCCGGGTTCGATTCCCGGCCGATGCACTGGTGCCACTAATAGAATCGTTTCAGAGCTATGCTGGAAA
fz3	2	AGTCAGAGGTGGGAGATGCTTGCACCAGCCGGGAATCGAACC
fz3	3	AGCATCTCCCACCTCTGACTGTTTCAGAGCTATGCTGGAAAC
fz3	4	TTTAACTTGCTATTTCTAGCTCTAAAACGAACGTGGAACAGCCCGGACTGCACCAGCCGGGAATCGAAC
fz4	1	GGCCCGGGTTCGATTCCCGGCCGATGCACGCCAGAAATCCCCGCCTTCGTTTCAGAGCTATGCTGGAAA
fz4	2	CATGGCCCTTGGAGTCCGGATGCACCAGCCGGGAATCGAACC
fz4	3	TCCGGACTCCAAGGGCCATGGTTTCAGAGCTATGCTGGAAAC
fz4	4	TTTAACTTGCTATTTCTAGCTCTAAAACAGCATCAGTTCCATGAAGGCTGCACCAGCCGGGAATCGAAC
smo	1	GGCCCGGGTTCGATTCCCGGCCGATGCATGAAGCACGTGCCCAAATGTGTTTCAGAGCTATGCTGGAAA
smo	2	CGCCGTTGATTTTTTCACACTGCACCAGCCGGGAATCGAACC
smo	3	GTGTGAAAAAATCAACGGCGGTTTCAGAGCTATGCTGGAAAC
smo	4	TTTAACTTGCTATTTCTAGCTCTAAAACGCACAAAGATCACTATGCAATGCACCAGCCGGGAATCGAAC
CG15744	1	GGCCCGGGTTCGATTCCCGGCCGATGCAGCGACGTCGACCGCAGCAGAGTTTCAGAGCTATGCTGGAAA
CG15744	2	AAGGTGTCTTTGTCCAGTTCTGCACCAGCCGGGAATCGAACC

CG15744	3	GAACTGGACAAAGACACCTTGTTTCAGAGCTATGCTGGAAAC
CG15744	4	TTTAACTTGCTATTTCTAGCTCTAAAACAATTTAGCCACTCACCCGATTGCACCAGCCGGGAATCGAAC
smog	1	GGCCCGGGTTCGATTCCCGGCCGATGCAAACATTATCCGAATCCACTGTTTCAGAGCTATGCTGGAAA
smog	2	CACGTGTCGGCTCATCGATGTGCACCAGCCGGGAATCGAACC
smog	3	CATCGATGAGCCGACACGTGGTTTCAGAGCTATGCTGGAAAC
smog	4	TTTAACTTGCTATTTCTAGCTCTAAAACTCCGTGCTGTTCCAGGCCGCTGCACCAGCCGGGAATCGAAC
CG31760	1	GGCCCGGGTTCGATTCCCGGCCGATGCACGGCGGCGAAAGGATCAAAGGTTTCAGAGCTATGCTGGAAA
CG31760	2	CAAGTTCTCGGTGGCAATGTTGCACCAGCCGGGAATCGAACC
CG31760	3	ACATTGCCACCGAGAACTTGGTTTCAGAGCTATGCTGGAAAC
CG31760	4	TTTAACTTGCTATTTCTAGCTCTAAAACCTCCATTCGTTAGATTCAGGTGCACCAGCCGGGAATCGAAC
CG12290	1	GGCCCGGGTTCGATTCCCGGCCGATGCATTTGGATAACGCCTGGCTTGGTTTCAGAGCTATGCTGGAAA
CG12290	2	TCTCCGCGCTCATCGATGGTTGCACCAGCCGGGAATCGAACC
CG12290	3	ACCATCGATGAGCGCGGAGAGTTTCAGAGCTATGCTGGAAAC
CG12290	4	TTTAACTTGCTATTTCTAGCTCTAAAACCGGCTGTTGGCGCTGATCAATGCACCAGCCGGGAATCGAAC
CG32447	1	GGCCCGGGTTCGATTCCCGGCCGATGCAATCGGATCGCAACGCCTACCGTTTCAGAGCTATGCTGGAAA
CG32447	2	TCATGAGCATGGTGAGGGTGTGCACCAGCCGGGAATCGAACC
CG32447	3	CACCCTCACCATGCTCATGAGTTTCAGAGCTATGCTGGAAAC
CG32447	4	TTTAACTTGCTATTTCTAGCTCTAAAACCCGCTATCCGGGTGTAGATCTGCACCAGCCGGGAATCGAAC
cg44153	1	GGCCCGGGTTCGATTCCCGGCCGATGCACCGCCGCCACCCTTTCAGCCGTTTCAGAGCTATGCTGGAAA
cg44153	2	GGGCACGCAGGTGTGTACCATGCACCAGCCGGGAATCGAACC
cg44153	3	TGGTACACACCTGCGTGCCCGTTTCAGAGCTATGCTGGAAAC
cg44153	4	TTTAACTTGCTATTTCTAGCTCTAAAACGCGTAGTCTCGTTTATTTCGTGCACCAGCCGGGAATCGAAC
boss	1	GGCCCGGGTTCGATTCCCGGCCGATGCAACGGAGAAAGCCATTACCAGGTTTCAGAGCTATGCTGGAAA
boss	2	TTGTTGGGGCCCTTGTGGTATGCACCAGCCGGGAATCGAACC
boss	3	TACCACAAGGGCCCCAACAAGTTTCAGAGCTATGCTGGAAAC
boss	4	TTTAACTTGCTATTTCTAGCTCTAAAACATCGAGTACGTCGTGCACTGTGCACCAGCCGGGAATCGAAC
CG32547	1	GGCCCGGGTTCGATTCCCGGCCGATGCATCGCCTGGGACCGGATGCGCGTTTCAGAGCTATGCTGGAAA
CG32547	2	CAGATCCGCGCGATGCTCGTTGCACCAGCCGGGAATCGAACC
CG32547	3	ACGAGCATCGCGCGGATCTGGTTTCAGAGCTATGCTGGAAAC
CG32547	4	TTTAACTTGCTATTTCTAGCTCTAAAACCTTCTCGCGATGCACGTCCATGCACCAGCCGGGAATCGAAC
ru	1	GGCCCGGGTTCGATTCCCGGCCGATGCACTAAGTGGGGCACCGTCACAGTTTCAGAGCTATGCTGGAAA
ru	2	CTGGTGGATCAGCACCCACCTGCACCAGCCGGGAATCGAACC

ru	3	GGTGGGTGCTGATCCACCAGGTTTCAGAGCTATGCTGGAAAC
ru	4	TTTAACTTGCTATTTCTAGCTCTAAAACGTTCTCTTGAATAGAGGGCATGCACCAGCCGGGAATCGAAC
rho	1	GGCCCGGGTTCGATTCCCGGCCGATGCAGAATGTAAACGAAACCAAGGGTTTCAGAGCTATGCTGGAAA
rho	2	GCCGATAGACCAGCACCGAATGCACCAGCCGGGAATCGAACC
rho	3	TTCGGTGCTGGTCTATCGGCGTTTCAGAGCTATGCTGGAAAC
rho	4	TTTAACTTGCTATTTCTAGCTCTAAAACGGGGACCCTTGGCGAAGGCGTGCACCAGCCGGGAATCGAAC
stet	1	GGCCCGGGTTCGATTCCCGGCCGATGCAGGCCATGGAGATCCTGCCCGGTTTCAGAGCTATGCTGGAAA
stet	2	AGGGTATGGGTCCCCTTGGCTGCACCAGCCGGGAATCGAACC
stet	3	GCCAAGGGGACCCATACCCTGTTTCAGAGCTATGCTGGAAAC
stet	4	TTTAACTTGCTATTTCTAGCTCTAAAACGATAGTTAGGCCAGCAATGGTGCACCAGCCGGGAATCGAAC
rho-4	1	GGCCCGGGTTCGATTCCCGGCCGATGCAGCGACAGGACACCATCGATAGTTTCAGAGCTATGCTGGAAA
rho-4	2	AGATCAGTTCCTTCAGCTCGTGCACCAGCCGGGAATCGAACC
rho-4	3	CGAGCTGAAGGAACTGATCTGTTTCAGAGCTATGCTGGAAAC
rho-4	4	TTTAACTTGCTATTTCTAGCTCTAAAACGCGACGGCACCGGACAAATGTGCACCAGCCGGGAATCGAAC
rho-5	1	GGCCCGGGTTCGATTCCCGGCCGATGCAGCAGCATGAGCTCCACGCGCGTTTCAGAGCTATGCTGGAAA
rho-5	2	GTCCGCAAACGGACCCGGAGTGCACCAGCCGGGAATCGAACC
rho-5	3	CTCCGGGTCCGTTTGCGGACGTTTCAGAGCTATGCTGGAAAC
rho-5	4	TTTAACTTGCTATTTCTAGCTCTAAAACATCACTATAGTTCGAAAGATTGCACCAGCCGGGAATCGAAC
rho-6	1	GGCCCGGGTTCGATTCCCGGCCGATGCAGAGAATTCCACGGATTCGGAGTTTCAGAGCTATGCTGGAAA
rho-6	2	TTCCGGTTTGAAGATCAGTATGCACCAGCCGGGAATCGAACC
rho-6	3	TACTGATCTTCAAACCGGAAGTTTCAGAGCTATGCTGGAAAC
rho-6	4	TTTAACTTGCTATTTCTAGCTCTAAAACGGGCACCCGCCACAATCGCTTGCACCAGCCGGGAATCGAAC
rho-7	1	GGCCCGGGTTCGATTCCCGGCCGATGCACCGGAGCTGGCTACCCCAGGGTTTCAGAGCTATGCTGGAAA
rho-7	2	GTGCCGCCGGATGTCTTGTTTGCACCAGCCGGGAATCGAACC
rho-7	3	AACAAGACATCCGGCGGCACGTTTCAGAGCTATGCTGGAAAC
rho-7	4	TTTAACTTGCTATTTCTAGCTCTAAAACGAACGAGAATCCCTTGAGGATGCACCAGCCGGGAATCGAAC
Lgr3	1	GGCCCGGGTTCGATTCCCGGCCGATGCATACGGCAGGAGCATCGCCGTGTTTCAGAGCTATGCTGGAAA
Lgr3	2	GATTCTCGTCGCTGCTGTCGTGCACCAGCCGGGAATCGAACC
Lgr3	3	CGACAGCAGCGACGAGAATCGTTTCAGAGCTATGCTGGAAAC
Lgr3	4	TTTAACTTGCTATTTCTAGCTCTAAAACCGGGCAGGTGGTGCAGATGGTGCACCAGCCGGGAATCGAAC
Lgr4	1	GGCCCGGGTTCGATTCCCGGCCGATGCAGGAAATTGCGGACGCCACAGGTTTCAGAGCTATGCTGGAAA
Lgr4	2	GATCGTGCTGCGGCAGTCGTTGCACCAGCCGGGAATCGAACC

Lgr4	3	ACGACTGCCGCAGCACGATCGTTTCAGAGCTATGCTGGAAAC
Lgr4	4	TTTAACTTGCTATTTCTAGCTCTAAAACGTTAGGAGCAGAGAGCTTAGTGCACCAGCCGGGAATCGAAC
rk	1	GGCCCGGGTTCGATTCCCGGCCGATGCAGGAGAACGTGATGCTGCCATGTTTCAGAGCTATGCTGGAAA
rk	2	TGGAACGACTGCGGTGGCAATGCACCAGCCGGGAATCGAACC
rk	3	TTGCCACCGCAGTCGTTCCAGTTTCAGAGCTATGCTGGAAAC
rk	4	TTTAACTTGCTATTTCTAGCTCTAAAACGGCTGCCCCTTATGGCCCAGTGCACCAGCCGGGAATCGAAC
Lgr1	1	GGCCCGGGTTCGATTCCCGGCCGATGCAAGCACCCGAGTCTGTCCCAGGTTTCAGAGCTATGCTGGAAA
Lgr1	2	ACTAACGAGGTGCTGGAGAGTGCACCAGCCGGGAATCGAACC
Lgr1	3	CTCTCCAGCACCTCGTTAGTGTTTCAGAGCTATGCTGGAAAC
Lgr1	4	TTTAACTTGCTATTTCTAGCTCTAAAACACGCGGTGAGTCCAAAGAAATGCACCAGCCGGGAATCGAAC
CG13575	1	GGCCCGGGTTCGATTCCCGGCCGATGCAAGCTCGAGTTGTACCTAGAAGTTTCAGAGCTATGCTGGAAA
CG13575	2	GCACGGGTCGCATCACGGCCTGCACCAGCCGGGAATCGAACC
CG13575	3	GGCCGTGATGCGACCCGTGCGTTTCAGAGCTATGCTGGAAAC
CG13575	4	TTTAACTTGCTATTTCTAGCTCTAAAACGTCGATGGGCTCGGAGTAGGTGCACCAGCCGGGAATCGAAC
CG12796	1	GGCCCGGGTTCGATTCCCGGCCGATGCATCGAAGCTCCCCGGAAGCGTGTTTCAGAGCTATGCTGGAAA
CG12796	2	CACAGAAGTCGGAGACGGCCTGCACCAGCCGGGAATCGAACC
CG12796	3	GGCCGTCTCCGACTTCTGTGGTTTCAGAGCTATGCTGGAAAC
CG12796	4	TTTAACTTGCTATTTCTAGCTCTAAAACCACAGAAGTACGGTAGCCAATGCACCAGCCGGGAATCGAAC

Table S3: Primers used for targeted genomic sequencing

Gene	#	Sequence
CG15614	1	TCGTCGGCAGCGTCAGATGTGTATAAGAGACAGCCGGTTGGCTAATTGGGCAGG
CG15614	2	GTCTCGTGGGCTCGGAGATGTGTATAAGAGACAGGCCGATGGCGTCATCTTGGG
CG15614	3	TCGTCGGCAGCGTCAGATGTGTATAAGAGACAGCGTGTCTGAGCCTGGATAGATTCATC
CG15614	4	GTCTCGTGGGCTCGGAGATGTGTATAAGAGACAGCTGTGACGTGACAAGTTCCCGAG
CG15614	5	TCGTCGGCAGCGTCAGATGTGTATAAGAGACAGggaaagggagtaagtggccatatgtt
CG15614	6	GTCTCGTGGGCTCGGAGATGTGTATAAGAGACAGCTATTAGCAGCGGGGTTGTTAGCC
CrzR	1	TCGTCGGCAGCGTCAGATGTGTATAAGAGACAGGGTGGTCAGCAATTGGTCCAC
CrzR	2	GTCTCGTGGGCTCGGAGATGTGTATAAGAGACAGCTATTGGTGTGCGCCAGTGAG
CrzR	3	TCGTCGGCAGCGTCAGATGTGTATAAGAGACAGCATTCGTGGAGGAGTTTTACCAGTGC
CrzR	4	GTCTCGTGGGCTCGGAGATGTGTATAAGAGACAGcaggttagtacgggaatgtaaactcc
CrzR	5	TCGTCGGCAGCGTCAGATGTGTATAAGAGACAGCGCCATCTTCTTCTTCGGCATG
CrzR	6	GTCTCGTGGGCTCGGAGATGTGTATAAGAGACAGcgcaaaaacaagtacgaaatgtggtg
mAchR-A	1	TCGTCGGCAGCGTCAGATGTGTATAAGAGACAGGGCTATACGATCCCTATTCGGGAATG
mAchR-A	2	GTCTCGTGGGCTCGGAGATGTGTATAAGAGACAGAGGATATCATCACCATGACATTGCCC
mAchR-A	3	TCGTCGGCAGCGTCAGATGTGTATAAGAGACAGCGGTGGTGAATCACGCCTCTG
mAchR-A	4	GTCTCGTGGGCTCGGAGATGTGTATAAGAGACAGgtcctacGCTTGACTTGCGC
mAchR-A	5	TCGTCGGCAGCGTCAGATGTGTATAAGAGACAGCGTACACCTGCTTCGGCAG
mAchR-A	6	GTCTCGTGGGCTCGGAGATGTGTATAAGAGACAGctacCTGGAGACGGAGCTTTGTAAAG
PDFR	1	TCGTCGGCAGCGTCAGATGTGTATAAGAGACAGCGATGTTCATTGTTCGCTTTGCAGC
PDFR	2	GTCTCGTGGGCTCGGAGATGTGTATAAGAGACAGGATTCAAATGTTCCGGCCGTCGG
PDFR	3	TCGTCGGCAGCGTCAGATGTGTATAAGAGACAGCCTACCACAAACAGGTACCTAAAGCC
PDFR	4	GTCTCGTGGGCTCGGAGATGTGTATAAGAGACAGCTGCGCGTATCTACGCCATG
PDFR	5	TCGTCGGCAGCGTCAGATGTGTATAAGAGACAGGACACCCTAAAAAGTGATTTCAAAGG
PDFR	6	GTCTCGTGGGCTCGGAGATGTGTATAAGAGACAGGCAGGGAAACTATAAGGGCGAACAG
Tre1	1	TCGTCGGCAGCGTCAGATGTGTATAAGAGACAGGCAGACATGCAGATGGATGAACCG
Tre1	2	GTCTCGTGGGCTCGGAGATGTGTATAAGAGACAGGCCTGTGCGGCAATTCAGCATAG
Tre1	3	TCGTCGGCAGCGTCAGATGTGTATAAGAGACAGGCTCGATTCCAACGACAGAGCTG
Tre1	4	GTCTCGTGGGCTCGGAGATGTGTATAAGAGACAGCTGCCAGGGGATTAGGGTGACGGTTG

Tre1	5	TCGTCGGCAGCGTCAGATGTGTATAAGAGACAGCAGATCGCAGCGGCCAAGGGCTC
Tre1	6	GTCTCGTGGGCTCGGAGATGTGTATAAGAGACAGCGAGGTATTGCGCTCGTCGTCCAC

References

1. J. O. Gummadova, G. A. Coutts, N. R. J. Glossop, Analysis of the Drosophila Clock promoter reveals heterogeneity in expression between subgroups of central oscillator cells and identifies a novel enhancer region. *J. Biol. Rhythms* **24**, 353–367 (2009).

2. B. Deng, *et al.*, Chemoconnectomics: Mapping Chemical Transmission in Drosophila. *Neuron* **101**, 876–893.e4 (2019).

3. L. Zhang, *et al.*, DN1(p) circadian neurons coordinate acute light and PDF inputs to produce robust daily behavior in Drosophila. *Curr. Biol.* **20**, 591–599 (2010).
